# Comparison of Isomerase and Weimberg Pathway for γ-PGA Production From Xylose by Engineered *Bacillus subtilis*

**DOI:** 10.3389/fbioe.2019.00476

**Published:** 2020-01-21

**Authors:** Birthe Halmschlag, Kyra Hoffmann, René Hanke, Sastia P. Putri, Eiichiro Fukusaki, Jochen Büchs, Lars M. Blank

**Affiliations:** ^1^Institute of Applied Microbiology, Aachen Biology and Biotechnology, RWTH Aachen University, Aachen, Germany; ^2^AVT-Biochemical Engineering, RWTH Aachen University, Aachen, Germany; ^3^Department of Biotechnology, Graduate School of Engineering, Osaka University, Osaka, Japan

**Keywords:** *Bacillus subtilis*, γ-PGA, online viscosity measurement, metabolic engineering, weimberg pathway, xylose, metabolome analysis

## Abstract

The production of poly-γ-glutamic acid (γ-PGA), a biopolymer consisting of D- and L-glutamic acid monomers, currently relies on L-glutamate, or citrate as carbon substrates. Here we aimed at using plant biomass-derived substrates such as xylose. γ-PGA producing microorganisms including *Bacillus subtilis* natively metabolize xylose via the isomerase pathway. The Weimberg pathway, a xylose utilization pathway first described for *Caulobacter crescentus*, offers a carbon-efficient alternative converting xylose to 2-oxoglutarate without carbon loss. We engineered a recombinant *B. subtilis* strain that was able to grow on xylose with a growth rate of 0.43 h^−1^ using a recombinant Weimberg pathway. Although ion-pair reversed-phase LC/MS/MS metabolome analysis revealed lower concentrations of γ-PGA precursors such as 2-oxoglutarate, the γ-PGA titer was increased 6-fold compared to the native xylose isomerase strain. Further metabolome analysis indicates a metabolic bottleneck in the phosphoenolpyruvate-pyruvate-oxaloacetate node causing bi-phasic (diauxic) growth of the recombinant Weimberg strain. Flux balance analysis (FBA) of the γ-PGA producing *B. subtilis* indicated that a maximal theoretical γ-PGA yield is achieved on D-xylose/ D-glucose mixtures. The results of the *B. subtilis* strain harboring the Weimberg pathway on such D-xylose/ D-glucose mixtures demonstrate indeed resource efficient, high yield γ-PGA production from biomass-derived substrates.

## Introduction

Poly-γ-glutamic acid (γ-PGA) is a biopolymer consisting of D- and L-glutamic acid monomers. The monomers are linked via amide linkages between the γ-carboxyl and the amino group of monomers γ-PGA is a non-toxic, biodegradable polymer. Due to these properties, γ-PGA is suitable for various applications in industrial fields including bioremediation, food sector, and medical use. The chemical synthesis of γ-PGA is complex since glutamate has two carboxyl groups. Thus, industrial production of this polymer is solely based on bacterial fermentation. The cost of production is hindering many applications of biopolymers (Kreyenschulte et al., 2014). Therefore, lowering the production cost by utilization of cheap substrates derived from plant biomass becomes an important task for a more efficient bioprocess of γ-PGA. One of the suitable substrates is xylose, the second most abundant carbohydrate in nature and the main pentose of hemicellulose in plant biomass. Depending on the origin of the hemicellulose, xylose makes up 90% of the hemicellulose (Saha, 2003).

The production of γ-PGA depends on the PGA synthetase catalyzing the polymerization reaction of L- and D-glutamic acid monomers and exporting the γ-PGA to the extracellular space. The glutamic acid precursors can be either *de novo* synthesized or imported from the medium. For *de novo* synthesis of glutamate from glucose, glucose is converted to acetyl-CoA by glycolysis, which then enters the TCA cycle to form 2-oxoglutarate. The glutamate synthase encoded by *gltAB* catalyzes the conversion of 2-oxoglutarate and glutamine to L-glutamate (Bohannon et al., 1985; Belitsky et al., 2000). This reaction is NADPH dependent. In absence of glutamine, L-glutamate can be synthesized from 2-oxoglutarate and ammonia by glutamate dehydrogenase. L-glutamate can be converted to D-glutamate directly by glutamic acid racemase or indirectly via D-amino acid aminotransferase (Ashiuchi, 2010).

**Figure 1:**
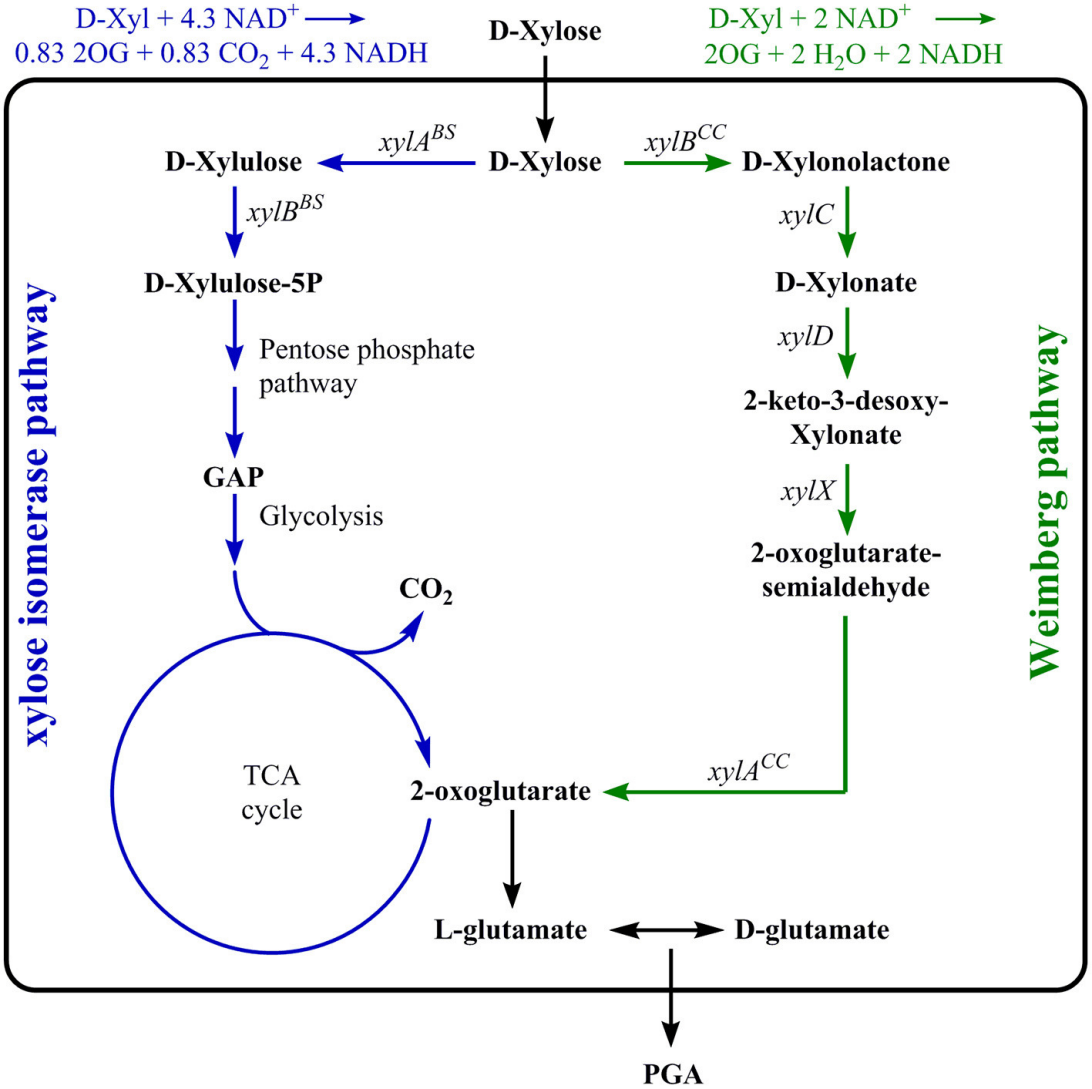
Metabolic pathways for γ-PGA production with xylose utilization. The isomerase pathway (blue) is natively present in *Bacillus subtilis*. The genes *xylA* and *xylB* encode the xylose isomerase and xylulokinase, respectively. These enzymes convert xylose to xylulose-5-phosphate, a pentose phosphate pathway (PPP) intermediate. The Weimberg pathway as present in *Caulobacter crescentus* (green) consists of five genes, *xylA, xylB, xylC, xylD*, and *xylX*. It is a linear pathway converting xylose to 2-oxoglutarate.

Besides glucose, *B. subtilis* 168 can also grow on xylose as sole carbon source. In *B. subtilis* 168, xylose is taken up via the arabinose transporter. Xylose is metabolized by a combination of the xylose isomerase pathway and the pentose phosphate pathway (PPP). The genes encoding the xylose isomerase and xylulokinase are *xylA* and *xylB*, respectively. These enzymes convert xylose to xylulose-5-phosphate, which is an intermediate of the PPP. In other bacteria as *Pseudomonas taiwanensis* VLB120 and *Caulobacter crescentus* the alternative Weimberg pathway for the conversion of xylose to 2-oxoglutarate has been described (Weimberg, 1961; Stephens et al., 2007; Köhler et al., 2015). The *C. crescentus xyl* operon including the five genes *xylXABCD* was shown to encode the enzymes for this linear D-xylose oxidative pathway. D-xylose is converted via D-xylono-1,4-lactone, xylonate, 2-keto-3-deoxy-D-xylonate, and 2-oxoglutarate-semialdehyde to 2-oxoglutarate (Stephens et al., 2007). The Weimberg pathway was successfully integrated into *Pseudomonas putida* S12 to enable the utilization of xylose as carbon source (Meijnen et al., 2009). Whereas, for the wild-type strain xylose is catabolized via the PPP, the integration of the Weimberg pathway to *B. subtilis* and simultaneous deletion of the native xylose degradation pathway likely directs the carbon flux to 2-oxoglutarate with a theoretical carbon yield of 100% (see Figure 1).

In this study, γ-PGA production with xylose as substrate is demonstrated. The core stoichiometric model for growth and γ-PGA formation (Zhu et al., 2013) was extended with metabolic pathways for xylose utilization. The model was used to calculate the γ-PGA production rate and yield for different substrate compositions. Metabolite measurements were carried out to validate the hypothesized higher precursor supply for xylose metabolism via the Weimberg pathway. Using the engineered strains, γ-PGA was produced with xylose as sole carbon source as well as with glucose/xylose mixtures. In summary, we present an alternative γ-PGA production process that can be the basis for future cost efficient and eco-friendly γ-PGA production.

## Materials and Methods

### Reagents

D-glucose, L-glutamic acid, NH_4_Cl, K_2_HPO_4_, MgSO_4_·7 H_2_O, CaCl_2_·2 H_2_O, MnSO_4_·H_2_O, FeCl_3_·6 H_2_O, ZnSO_4_·7 H_2_O, Na_2_-EDTA, CuSO_4_·5 H_2_O, and CoCl_2_·6 H_2_O were purchased from Carl Roth GmbH+Co. KG (Karlsruhe, Germany). LC/MS-grade ultra-pure water, HPLC-grade chloroform, acetic acid, H_2_SO_4_, and NH_4_HCO_3_ were purchased from Wako Pure Chemical Industries, Ltd. (Osaka, Japan). 10-Camphorsulfonic acid and tributylamine were purchased from SigmaAldrich (MO, USA).

### Strains, Plasmids, and Growth Conditions

The bacterial strains and plasmids that were developed and used in this study are listed in Table 1. All cloning steps were carried out in *Escherichia coli* DH5α. The recombinase positive *E. coli* strain JM101 was used to obtain plasmids for the transformation of *B. subtilis*. For plasmid construction and counterselection, all strains were cultivated at 37°C in lysogeny broth (LB) medium containing 100 μg mL^−1^ spectinomycin or 0.5% (w/v) mannose as needed. For γ-PGA production and metabolome analysis, the *B. subtilis* strains were cultured in glucose minimal medium. The media compositions are described in detail in following paragraphs.

**Table 1:** Strains and plasmids used in this study.

**Strain/plasmid**	**Genotype/properties**	**Reference/source**
**STRAINS**		
*Escherichia coli* DH5α	*fhuA*2 Δ (*argF-lacZ*)U169 *phoA glnV*44 Φ80 Δ(*lacZ*)M15 *gyrA*96 *recA*1 *relA*1 *endA*1 thi-1 hsdR17	Meselson and Yuan, 1968
*Escherichia coli* JM101	*glnV*44 thi-1 Δ(*lac-proAB*) F′[*lacI^*q*^ZΔM15 traD*36 *proAB*^+^]	Messing et al., 1981
*Bacillus subtilis* Δspo	ΔSPβ Δ*skin* ΔPBSX ΔproΦ1 Δ*pks*::CmR, ΔproΦ3 *trp*+ Δ*manPA*::*erm* Δ*bpr* Δ*sigG* Δ*sigE* Δ*spoGA*	Halmschlag et al., 2019
*Bacillus subtilis* Δ*xylAB*	Δspo derivative; Δ*xylAB*	This study
*Bacillus subtilis* Δ*xylAB*::*xylXACD*^CC^	Δspo derivative; Δ*xylAB*::*xylXACD*^CC^	This study
*Bacillus subtilis* WB	Δspo derivative; Δ*xylAB*::*xylXACD*^CC^ Δgdh::P43-*xylB*^CC^	This study
*Bacillus subtilis* WB PX43-*pgs*	WB derivative; ΔP(pgs)::PX43	This study
*Bacillus subtilis* Ref PX43-*pgs*	Δspo derivative; ΔP(pgs)::PX43	This study
**PLASMIDS**		
pJOE-8739	Vector backbone	Wenzel and Altenbuchner, 2015
pBs-17	Deletion of *xylAB* genes	This study
pBs-19	Integration of P43 promoter into *gdh* locus	This study
pBs-24	Integration of *xylXACD* from *Caulobacter crescentus* into *xylAB* locus	This study
pBs-34	Integration of *xylB* from *C. crescentus* into *gdh* locus	
pBs-43	Integration of PX43 promoter upstream of *pgs*	This study

### Plasmid Construction

The plasmid pBs-17 was constructed to delete *xylAB* in *B. subtilis*. For pBs-17 construction, two target sequences (TS1 and TS2) located upstream and downstream of *xylAB* were amplified from *B. subtilis* genomic DNA using primer pairs BS-85/86 and BS-87/88 (primer listed in Supplementary Table 1). The target sequences were integrated into linearized pJOE-8739 by NEBuilder HiFi DNA Assembly as described by the manufacturer (New England Biolabs, Germany). pJOE-8739 was linearized using primers BS-25/26. For integration of *xylXACD*^*CC*^ and simultaneous deletion of *xylAB*^*BS*^, the previously constructed pBs-17 was linearized with BS-155 and BS-92. The codon-optimized *xylXA* and *xylCD* genes were cloned into pJET vectors and amplified with primer pairs BS-156/157 and BS-158/159, respectively. *xylXA* and *xylCD* were assembled with the linearized pBs-17 to obtain plasmid pBs-24. Plasmid pBs-19 was constructed to integrate the P43 into the *B. subtilis gdh* locus. The plasmid was constructed by integration of TS1, promoter P43, and TS2, which were amplified using primer pairs BS-99/100, BS-101/102, and BS-103/104, respectively, into linear pJOE-8739. Plasmid pBs-34 was constructed to integrate *xylB*^*CC*^ under control of the P43 promoter into the *B. subtilis gdh* locus. For construction of pBs-34, the previously constructed vector pBs-19 containing the TS1 for integration at the *gdh* site and the P43 promoter was linearized using primers BS-224/BS-225. The *xylB* gene and TS2 were amplified with BS-226/227 and BS-228/229, respectively. γ-PGA production was enabled by pBs-43. The native *B. subtilis* promoter Pxyl was fused with a CcpA binding site building promoter PX43. The PX43 *cre* site is natively used for expression control of *rbsR* and *yncC* in *B. subtilis* (Marciniak et al., 2012). The vector contains target sequences for the integration of the promoter upstream of the *pgs* operon (see Supplementary Information). The assembled plasmids were transformed into chemically competent *E. coli* DH5α. After analyzing the plasmids by PCR, correctly constructed plasmids were sequenced and retransformed to *E. coli* JM101 to obtain the plasmids for *B. subtilis* transformation.

### Strain Development

For the *Bacillus* transformations, naturally competent cells were prepared according to the Paris method (Harwood, 1990). The *xylAB* genes were deleted in *B. subtilis* Δspo using the plasmid pBs-17, which resulted in the development of the *B. subtilis* Δ*xylAB* strain. The integration of the Weimberg pathway was carried out by integration of *xylXACD* into *B. subtilis* Δspo using plasmid pBs-24. The resulting *B. subtilis* Δ*xylAB*::*xylXACD*^CC^ strain was transformed with pBs-34 to integrate *xylB*^CC^ into the *gdh* locus. The γ-PGA production with the native *B. subtilis pgs* operon was enabled by integrating the promoter PX43 resulting in *B. subtilis* WB PX43-pgs and *B. subtilis* Ref PX43-pgs for the strain containing the Weimberg pathway and for the reference strain (*B. subtilis* Δspo) that harbors the xylose isomerase pathway, respectively. All genome editing steps were performed using the markerless gene deletion system as described by Wenzel and Altenbuchner (2015).

### Flux Balance Model

The basic model for metabolic reactions required for γ-PGA synthesis was taken from Zhu et al. (2013). The model represents the central carbon metabolism including glycolysis, TCA cycle, and PPP. The included reaction for biomass formation is derived from Dauner and Sauer (2001). The biomass formation was modeled as a combination of eight precursors (3-phosphoglycerate, phosphoenolpyruvate, ribose-5-phosphate, erythose-4-phosphate, pyruvate, acetyl-CoA, 2-oxoglutarate, and methyl-tetrahydrofolate) and utilization of ATP and NAD(P)H. The production of γ-PGA as an ATP-dependent process is included in the model. The previously described metabolic model for γ-PGA production with *B. subtilis* (Zhu et al., 2013) was augmented with the two metabolic pathways for xylose utilization. The two reactions of the xylose isomerase pathway, xylose isomerase and xylulokinase were integrated. The heterologous pathway for the conversion of xylose to 2-oxoglutarate referred to as Weimberg pathway is added as described by Stephens et al. (2007). The resulting network consists of 48 reactions and 41 metabolites. For the calculation of γ-PGA production rates, a growth rate of 0.6 h^−1^ and an ATP maintenance coefficient of 9.9 mmol ${\text{g}}_{\text{cdw}}^{-1}$ h^−1^ as stated for the genome-scale model of *B. subtilis* by Oh et al. (2007) were used. The substrate uptake rate was fixed to 20 mmol ${\text{g}}_{\text{cdw}}^{-1}$ h^−1^ in total whereas the actual substrate uptake rate and by-product formation rates depend on the exact cultivation conditions and have to be determined. Based on these values, the γ-PGA production rate was calculated by flux balance analysis (FBA) for different substrate compositions with the γ-PGA production rate as objective function. Flux balance analysis was carried out using the Cobra Toolbox (Schellenberger et al., 2011) and Matlab (The MathWorks, Inc.,).

### Cultivation for Metabolite Analysis

For the cultivation of *B. subtilis* either xylose or xylonate was used as carbon source in a minimal salt medium for the main cultures. The minimal medium contained: 20 g Xylose, 7 g NH_4_Cl, 0.5 g KH_2_PO_4_, 0.5 g MgSO_4_, 0.2 g L-glutamic acid, 0.15 g CaCl_2_, 0.1 g MnSO_4_, 0.04 g FeCl_3_, and 1 mL of a trace element solution per liter. The trace element solution [according to Wenzel et al. (2011)] was modified and contained: 0.54 g ZnSO_4_·7 H_2_O, 30.15 g Na_2_-EDTA, 0.48 g CuSO_4_·5 H_2_O, and 0.54 g CoCl_2_·6 H_2_O per liter. The medium was buffered using 0.1 M potassium phosphate at pH 7. To obtain xylonate as carbon source, xylose was dissolved in 0.2 M potassium phosphate buffer (pH 7) to a concentration of 40 g L^−1^. *Pseudomonas putida* KT2440, which can convert xylose to xylonate, but is not able to grow on xylose/xylonate, was grown in LB medium overnight. Subsequently, the cells were centrifuged (5,000 g, 10 min, RT) and added to the xylose solution. The mixture of xylose and cells was incubated for 16 h to allow the conversion of xylose to xylonate. Afterwards, the cells were removed by centrifugation and the solution was sterile filtered. By addition of the other components, the medium was created as described above with xylonate as C-source instead of xylose. For preculturing, the *Bacillus* cells were cultivated in LB medium overnight and then transferred to fresh minimal medium containing the same ingredients as the main culture medium. The LB medium contained 10 g L^−1^ tryptone, 5 g L^−1^ yeast extract, and 10 g L^−1^ NaCl and had a pH of 7.4. The main cultures were inoculated to an OD_600_ of 0.1 from the preculture. Except for the trace element solution, which was sterile filtered, all the media components were sterilized by autoclaving for 20 min at 121°C. The cultivations were carried out in 250 mL Erlenmeyer flasks containing 25 mL medium. The cultures were incubated on a rotary shaker with a 25 mm shaking diameter at 37°C and 200 rpm (Bio-Shaker BR-3000LF, Taitec, Saitama, Japan). All the cultivations were performed in triplicate, and the data are presented as the mean. The cell growth was monitored by OD_600_ measurements of samples obtained from the shake flasks using the GeneQuant 100 spectrophotometer (GE Healthcare UK Ltd., Buckinghamshire, UK).

### Cultivation for On-Line Monitoring (OTR and Viscosity Measurements)

On-line monitoring of the main cultures was realized using an in-house manufactured Respiration Activity Monitoring System (RAMOS) recording the oxygen transfer rate (OTR) (Anderlei and Büchs, 2001; Anderlei et al., 2004). Commercial versions of this system are available from Kuhner AG, Birsfelden, Switzerland or HiTech Zang, Herzogenrath, Germany.

For main cultures in shake flasks, a master mix was prepared by inoculating the mineral medium with the pre-culture. Subsequently, the desired filling volume was transferred to RAMOS flasks and to additional Erlenmeyer flasks with cotton plugs for sampling and off-line analysis. Main cultures were run under the following conditions: 250 mL shake flasks, filling volume 20 mL, shaking frequency 200 rpm, shaking diameter 50 mm, temperature 37°C, initial OD_600_ = 0.1. RAMOS flasks and sampling flasks were filled from the same master mix and cultivated in parallel and under identical conditions to guarantee that cultures ran synchronously. Erlenmeyer flasks withdrawn for sampling were not placed back on the shaker.

For online viscosity measurement a new device according to Sieben et al. (2019) was used. It evaluates the shift in the angular position of the bulk liquid relative to the direction of the centrifugal acceleration. The measurement system is different from the previously used, based on power input measurement (Regestein Née Meissner et al., 2017).

### Batch Fermentations

The batch fermentations were carried out in a stirred tank bioreactor (Eppendorf BioFlo120) equipped with a pH electrode, dissolved oxygen (DO)-electrode, a temperature sensor, two Rushton turbines, a sampling tube, and a cell growth quantifier for bioreactors (CGQ BioR; Aquila Biolabs, Baesweiler, Germany). The reactor was sterilized at 121°C for 20 min at 1 bar overpressure. The experiments were carried out at a temperature of 37°C with a filling volume of 0.5 L. As culture medium, the identical glucose minimal medium as used for the shake flask experiments was employed. The pH value was adjusted to 7 prior to inoculation and kept constant by the addition of 2 M HCl or 2 M NaOH throughout the fermentation. The aeration rate was set to 1 vvm. The cultivation was stirred with an agitation rate of 800 rpm. Prior to inoculation 0.5 mL L^−1^ antifoam was added to avoid foam formation. At time point t_0_ the reactor was inoculated with cells from a pre-culture to obtain an initial OD_600_ of 0.1. For this purpose, the required pre-culture volume was centrifuged (5,000 g, 5 min, 4°C), washed with 0.9% NaCl and resuspended in 3 mL 0.9% NaCl. The suspension was directly injected into the fermenter.

### Analysis of γ-PGA Concentration

The concentration of γ-PGA was determined by the photometric cetyltrimethylammonium bromide (CTAB) assay. CTAB binds to negatively charged molecules, in the case of γ-PGA forming a water-insoluble complex, resulting in an increased turbidity of the solution. Samples taken from shake flask or bioreactor fermentations were centrifuged (16,000 g, 60 min, 4°C). The sample supernatant was stored at −20°C until analyzed. Based on a calibration curve using standards of 1 MDa γ-PGA in the range of 0.1–10 μg mL^−1^, the γ-PGA from the culture supernatant was quantified by measuring the turbidity at 400 nm. For this purpose, 100 μL 0.7 M CTAB in 2% NaOH were added to 100 μL sample or standard solution in a 96-well microtiter plate. After 1 min of incubation the turbidity was measured in a Synergy MX microplate reader (BioTek Instruments, Winooski, USA).

### Sample Preparation for Metabolome Analysis

A sample volume satisfying the equation: sampling volume (mL) × OD_600_ = 5 was filtered with a PVDF filter (pore size 0.45 μm) using vacuum. The filter was washed with the doubled amount of 300 mM NH_4_HCO_3_ (1:2 ratio, sample: NH_4_HCO_3_) solution. Subsequently, the metabolism was quenched by soaking the filter in liquid N_2_. The filter was transferred to a 2 mL sample tube and was stored at −80°C until metabolite extraction. To extract the metabolites, 1.875 mL of extraction solvent (1 H_2_O:2 MeOH:2 chloroform, including 7 nM 10-camphorsulfonic acid as internal standard) was added to the sample tube including the filter. After vortexing, the sample tube was centrifuged at 16,000 g for 3 min at 4°C. Three hundred fifty microlitre of the supernatant were transferred to a 1.5 mL sampling tube, concentrated by vacuum centrifugation for 2 h and freeze dried overnight. The pellet was stored at −80°C until analyzed by LC-MS.

### Ion-Pair-LC/MS/MS Analysis

The dried sample was resuspended in 50 μL ultra-pure water and was transferred into a conical glass vial. The ion-pair-liquid chromatography coupled with tandem mass spectrometry (LC/MS/MS) analysis was performed using a Shimadzu Nexera UHPLC system coupled with an LCMS 8030 Plus device (Shimadzu Co., Kyoto, Japan). The system was equipped with a PE capped CERI L-column 2ODS column (2.1 × 150 mm, particle size 3 mm, Chemicals Evaluation and Research Institute, Tokyo, Japan). As mobile phase a gradient of a mixture of solvent A and B was used, where solvent A is 10 mM tributylamine and 15 mM acetate in ultra-pure water and solvent B was pure methanol. The flow rate was set to 0.2 mL min^−1^. For gradient elution of metabolites, starting from 0% concentration of solvent B, the concentration of B was increased to 15% after 1 min with a gradient of 30% min^−1^, hold for 1.5 min, increased to 50% within 5 min and subsequently increased to 100% within 2 min. The 100% solvent B concentration was held for 1.5 min, decreased to 0% from 11.5 min on and held at this concentration for 8.5 min. The column oven temperature was set to 45°C. The MS parameters were as follows: probe position, 1.5 mm; desolvation line temperature, 250°C; drying gas flow, 15 L/min; heat block temperature, 400°C; and nebulized gas flow, 2 L min^−1^. As blank sample, a filter washed with NH_4_HCO_3_ without biomass addition was used for extraction of compounds that are not of biological origin. As a Quality Control (QC) sample, 2 μL of each analyzed sample of a batch were pooled into a vial. For each sample (including the QC sample), 3 μL were injected to the ion-pair-LC/MS/MS for metabolite analysis.

### Data Processing and Analysis

The calculation of the peak area was carried out using MRMPROBS ver. 2.38 and checked manually. Peaks that are not of biological origin as identified with the blank sample were excluded. The data was normalized according to the peak area of the internal standard, 10-camphorsulfonic acid. SIMCA 13 (Umetrics, Umeå, Sweden) was used for principal component analysis (PCA).

## Results

### Metabolic Pathways for Xylose Utilization and Growth of Engineered Strains

Stoichiometric comparison of the xylose isomerase pathway (Figure 1, blue) and the Weimberg pathway (green) suggested the benefit of the Weimberg pathway for γ-PGA production using xylose as carbon source. The conversion of xylose to 2-oxoglutarate via the Weimberg pathway theoretically results in 1 mol 2-oxoglutarate per mol xylose. On the contrary, the xylose isomerase pathway yields 0.83 mol 2-oxoglutarate per mol xylose due to the formation of CO_2_ in the TCA cycle. However, the amount of produced redox equivalents is higher if xylose is metabolized via the xylose isomerase pathway.

Based on the stoichiometric comparison, the insertion of the Weimberg pathway is hypothesized to result in higher precursor concentrations for γ-PGA synthesis. Therefore, the native xylose isomerase pathway encoded by the genes *xylAB* coding for the xylose isomerase and xylulokinase, was replaced by the genes *xylXABCD* originating from *C. crescentus*. As a negative control, the deletion mutant *B. subtilis* Δ*xylAB* not capable of growing on xylose was used. To test the effect of the deletion of the *xylAB* genes in *B. subtilis* and the subsequent integration of the Weimberg pathway, the strains *B. subtilis* Δspo (reference with isomerase pathway), *B. subtilis* Δ*xylAB* (negative control), *B. subtilis* Δ*xylAB*::*xylXACD*^CC^, and *B. subtilis* WB (Δ*xylAB*::*xylXACD*^CC^ Δ*gdh*::*xylB*) were grown on xylose and xylonate as substrates (Table 2).

**Table 2:** Growth of engineered *B. subtilis* strains on xylose and xylonate.

**Strain**	**Growth rate [h**^****−1****^**]**	
	**Xylonate**	**Xylose**
*Bacillus subtilis* Δspo	n.d.	0.22 ± 0.02
*Bacillus subtilis* Δ*xylAB*	no growth	No growth
*Bacillus subtilis* Δ*xylAB*::*xylXACD*^CC^	0.47 ± 0.01	No growth
*Bacillus subtilis* WB	n.d.	0.43 ± 0.01

*Bacillus subtilis Δspo encodes the isomerase pathway. Bacillus subtilis ΔxylAB was used as negative control without any catabolic pathway for xylose metabolism. Bacillus subtilis WB contains the genes encoding the Weimberg pathway. n.d., not determined*.

The initial strain *B. subtilis* Δspo is capable of growing on xylose as sole carbon. The growth rate on xylose was 0.22 h^−1^ without the addition of arabinose. For the laboratory strain *B. subtilis* 168, the addition of arabinose is reported to be required for the expression of *araE*. AraE is a transport protein of the major facilitator superfamily responsible for the uptake of pentoses including both arabinose and xylose. The deletion of *xylAB* resulted in the abolishment of growth on xylose as well as on xylonate. Since xylonate was commercially not available as carbon source, it was obtained from xylose conversion by the *P. taiwanensis* VLB120 glucose dehydrogenase. Therefore, the xylonate substrate contained xylose impurities. For this reason the growth rate of the reference strain and the Weimberg mutant was not tested on xylonate. For the mutant harboring the incomplete Weimberg pathway *B. subtilis* Δ*xylAB*::*xylXACD*^CC^ the growth rate on xylonate was 0.47 h^−1^. This strain is not able to grow on xylose as sole carbon source, as it lacks a functional xylose dehydrogenase (XDH) catalyzing the first step of the Weimberg pathway. The XDH converts xylose to xylonolactone. In other bacteria such as *P. putida*, the integration of the four genes *xylX, xylA, xylC*, and *xylD* was sufficient to enable growth on xylose, since the inherent glucose dehydrogenase (GDH) also accepts xylose as substrate (Meijnen et al., 2009). However, the *B. subtilis* GDH does not catalyze the conversion of xylose (Fujita et al., 1977). Therefore, the ability of *B. subtilis* to grow on xylose was only restored in Δ*xylAB* when all five genes of the Weimberg pathway were integrated into the genome. As shown in Table 2, the Weimberg pathway mutant exhibits a significantly higher growth rate (0.43 h^−1^) than the reference strain (0.22 h^−1^) on xylose.

### Measurement of Intracellular Precursor Supply

**Figure 2:**
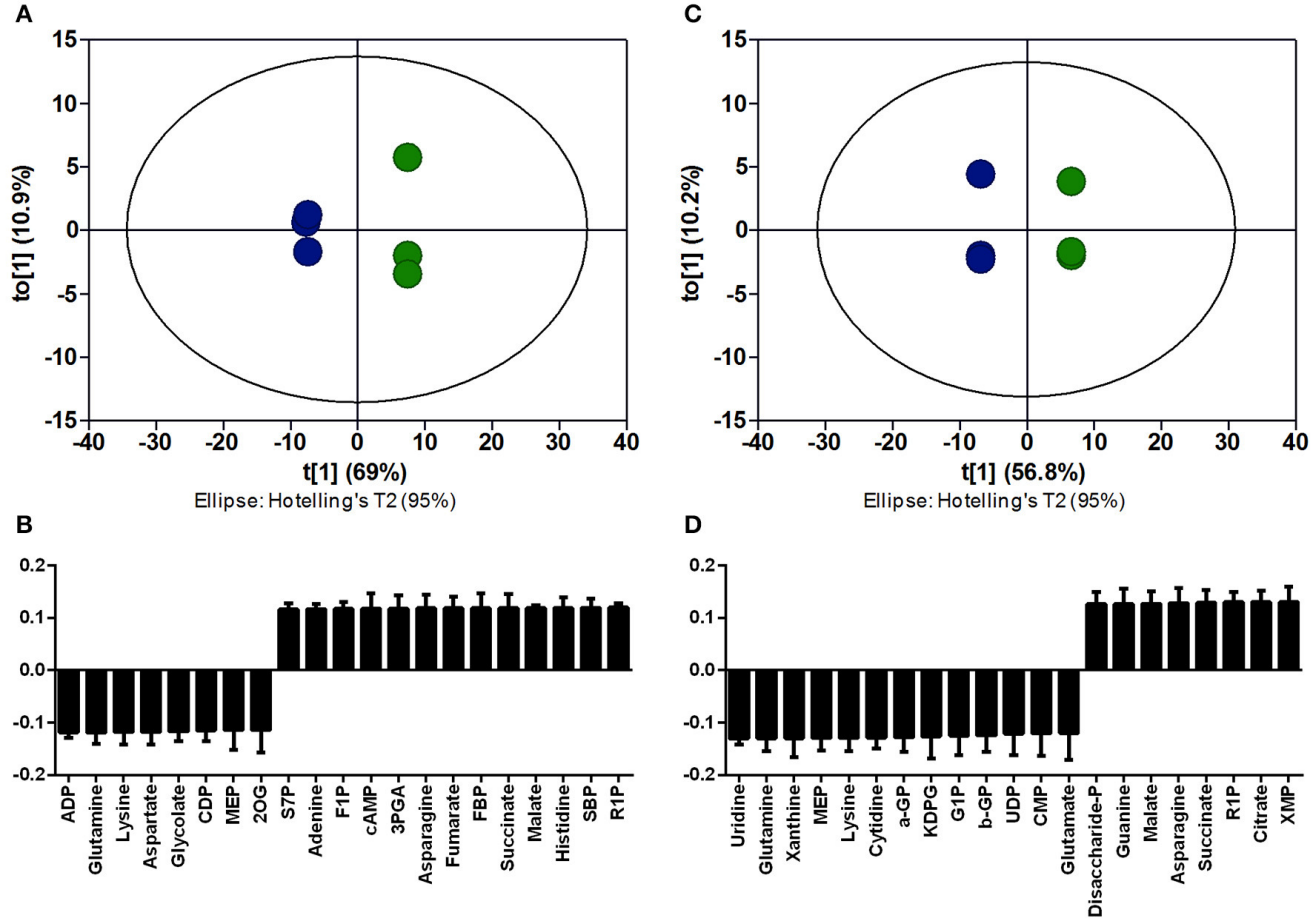
Orthogonal partial least squares discriminant analysis (OPLS-DA) for intracellular metabolites of *B. subtilis* WB in comparison to *B. subtilis* Δspo (reference) without **(A,B)** and with **(C,D)** γ-PGA production. The score plot for triplicate metabolome samples of the reference (blue) and *B. subtilis* WB (green) strains without **(A)** and with **(C)** γ-PGA production is given. The loading values without **(B)** and with **(D)** γ-PGA production for metabolites highly contributing to the separation are presented. High loading values correspond to increased metabolite concentrations for *B. subtilis* WB (PX43-*pgs*). 2OG, 2-oxoglutarate; 3PGA, 3-Phosphoglycerate; ADP, Adenosine 5′-diphosphate; a-GP, α-glycerophosphate; b-GP, β-glycerophosphate; cAMP, 3′,5′-cyclic AMP; CDP, cytidine 5′-diphosphate; CMP, cytidine 5′-monophosphate; CTP, cytidine 5′-triphosphate; DHAP, dihydroxyacetone phosphate; F1P, fructose-1-phosphate; FBP, fructose-bisphosphate; G1P, glucose-1-phosphate; GAP, D-glyceraldehyde 3-phosphate; GDP, guanosine 5′-diphosphate; KDPG, 2-keto-3-deoxy-6-phosphogluconate; MEP, methyerythritol 4-phosphate; PEP, phosphoenolpyruvate; R1P, ribose-1-phosphate; R5P, ribose-5-phosphate; S7P, sedoheptulose-7-phosphate; SBP, sedoheptulose-1,7-bisphosphate; UDP, uridine 5′-diphosphate; XMP, xanthosine 5′-phosphate.

**Figure 3:**
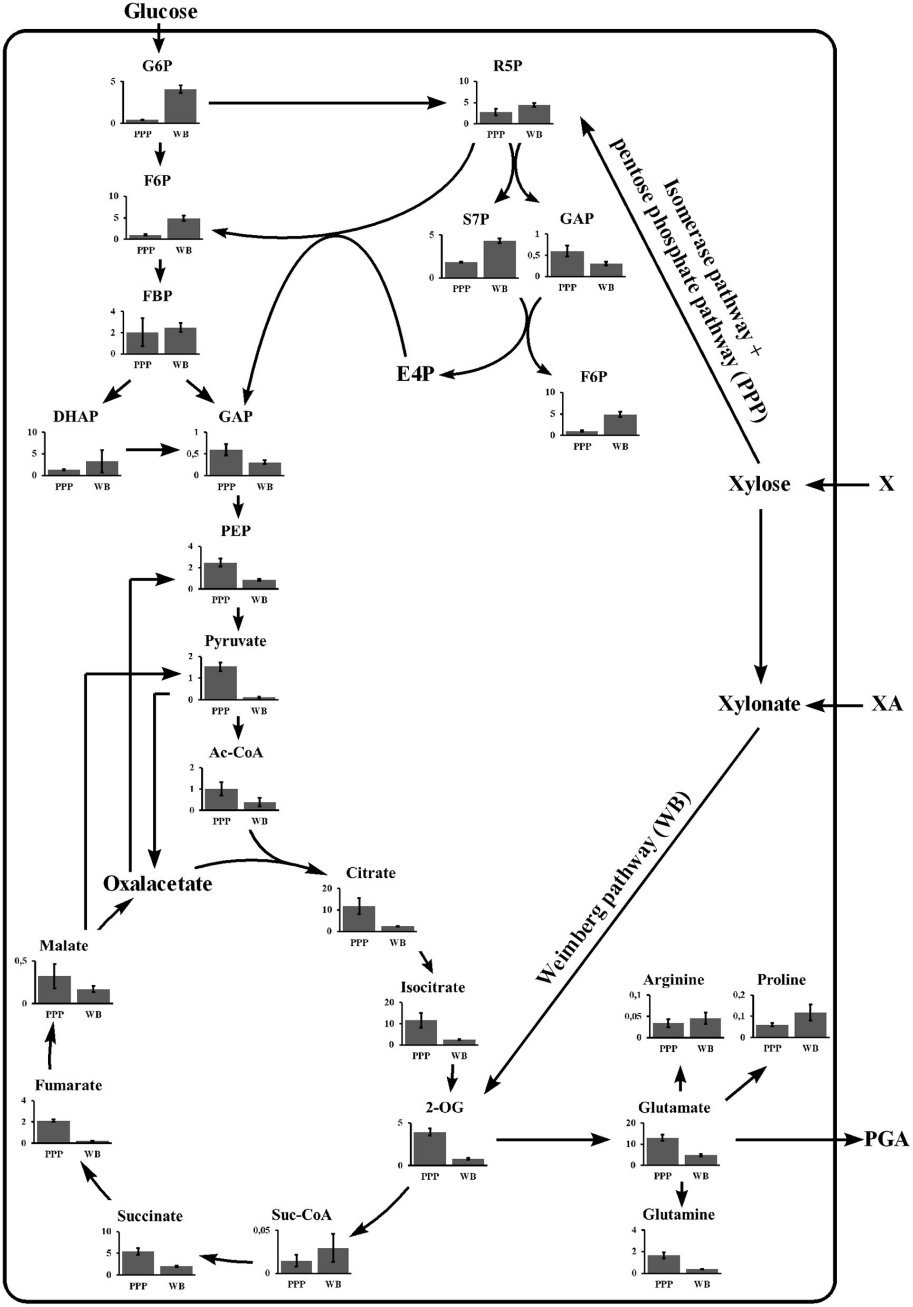
Intracellular metabolite analysis for *B. subtilis* WB and *B. subtilis* Δspo growing on xylose. The metabolite concentrations for glycolysis, PPP, TCA cycle, and glutamate derived amino acids are given for *B. subtilis* Δspo (PPP) and *B. subtilis* WB (WB). Cultivations were carried out in triplicates. The bar graphs represent the mean. The error bar indicates the standard deviation. 2OG, 2-oxoglutarate; DHAP, dihydroxyacetone phosphate; F6P, fructose-6-phosphate; FBP, fructose-bisphosphate; G6P, glucose-6-phosphate; GAP, D-glyceraldehyde 3-phosphate; R5P, ribose-5-phosphate; S7P, sedoheptulose-7-phosphate.

Since xylose is directly converted to 2-oxoglutarate, a higher precursor supply for γ-PGA production was hypothesized for growth utilizing the Weimberg pathway. For the comparison of the precursor supply in *B. subtilis* WB and the reference strain with and without γ-PGA production, metabolome analysis using ion pair LC/MS/MS was performed. To enable γ-PGA production on xylose as carbon source, a xylose inducible promoter fused with a catabolite response element (cre) site as it is present in the genome of *B. subtilis* (Marciniak et al., 2012) were integrated to control the expression of *pgs* in *B. subtilis* Ref PX43-*pgs* and *B. subtilis* WB PX43-*pgs*. Ninety eight metabolites, including central metabolites, amino acids, nucleotides, and cofactors, were annotated in the four strains *B. subtilis* Ref, *B. subtilis* Ref PX43-*pgs, B. subtilis* WB, and *B. subtilis* WB PX43-*pgs* (Supplementary Table 2). The metabolic profiles of these four strains were subjected to principal component analysis (PCA, Figure 2). PCA of the datasets revealed lower concentrations for intermediates of the TCA cycle, especially 2-oxoglutarate for *B. subtilis* WB in comparison to the reference catabolizing xylose through the xylose isomerase pathway (Figures 2, 3). In contrast the concentrations for intermediates of glycolysis and PPP as represented by high loading values for ribose-1-phosphate, D-glyceraldehyde 3-phosphate (GAP), or fructose-bisphosphate (FBP) were increased for growth via the Weimberg pathway. The results indicate a higher TCA cycle flux in the Weimberg pathway mutant, which is in accordance with the highly increased growth rate for *B. subtilis* WB. In turn, fluxes in gluconeogenesis and PPP are likely reduced in this strain, resulting in higher concentrations of the intermediates. For the production of γ-PGA, the utilization of the Weimberg pathway is favorable as the higher flux through the 2-oxoglutarate results in higher γ-PGA production.

**Figure 4:**
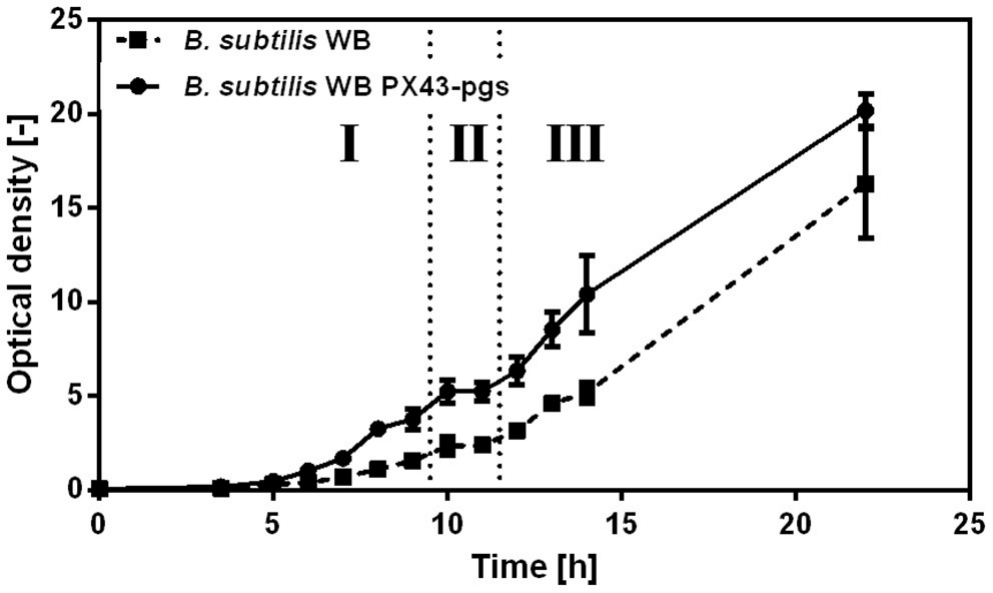
Growth of *B. subtilis* WB on xylose. Samples for metabolome analysis were taken in the three indicated growth phases. The plateau was observed for *B. subtilis* WB and *B. subtilis* WB PX43-*pgs*, thus independent from γ-PGA production. Cultivations were carried out in triplicates. Data represents the mean of biological triplicates. The error bar indicates the standard deviation.

**Figure 5:**
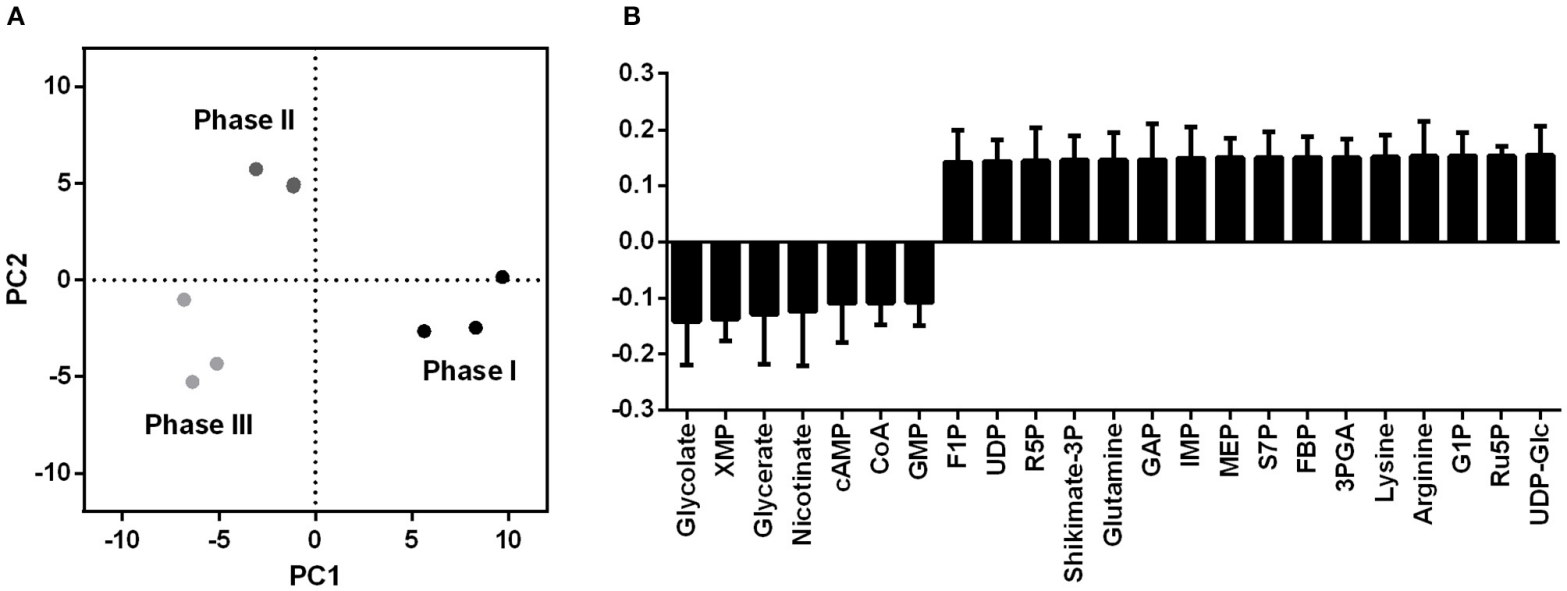
Principal component analysis (PCA) for different growth phases of *B. subtilis* WB PX43-*pgs* on xylose. The score plot **(A)** demonstrates the separation according to principal component 1 (PC1) and PC2 for triplicate samples obtained from three cultivation time points corresponding to the phases indicated in Figure 4. Samples were taken after 9, 11, and 14 h for phase I, II, and III, respectively. The loading data for metabolites exhibiting the highest values for PC1, thus changing throughout the growth phases, are shown in **(B)**. 3PGA, 3-phosphoglycerate; AMP, adenosine 5′-monophosphate; cAMP, 3′,5′-cyclic AMP; CoA, coenzyme A; F1P, fructose-1-phosphate; FBP, fructose-bisphosphate; G1P, glucose-1-phosphate; GAP, D-glyceraldehyde 3-phosphate; GMP, guanosine 5′-monophosphate; IMP, inosine 5′-monophosphate; MEP, methyerythritol 4-phosphate; R5P, ribose-5-phosphate; Ru5P, ribulose-5-phosphate; S7P, sedoheptulose-7-phosphate; UDP, uridine 5′-diphosphate; UDP-Glc, uridine 5′-diphosphate-glucose; XMP, xanthosine 5′-phosphate.

**Figure 6:**
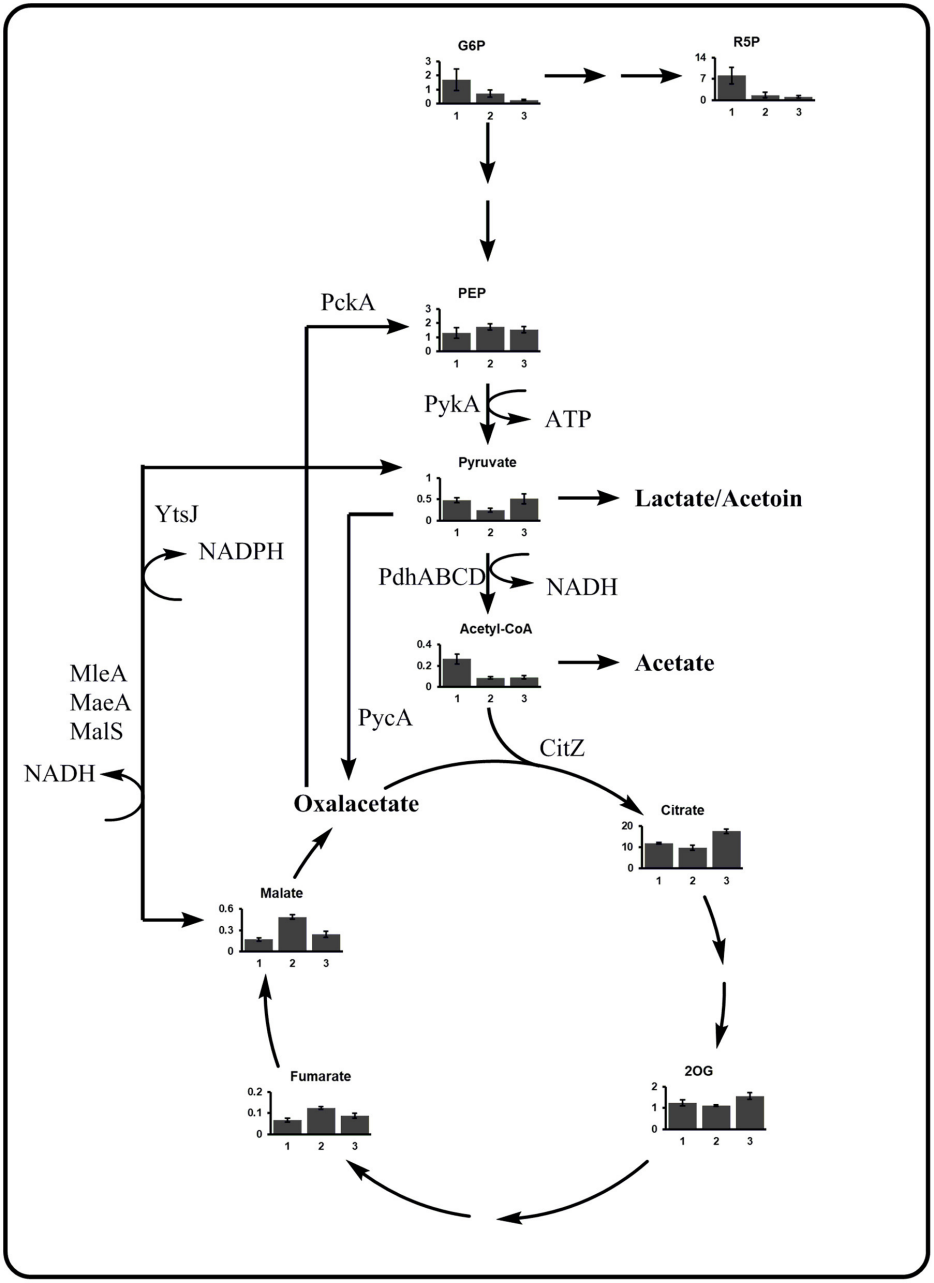
Metabolic profile of the PEP-pyruvate-oxaloacetate node for *B. subtilis* WB growing on xylose. The metabolite concentrations from the central carbon metabolism for the three growth phases as indicated in 4 are given. Samples were taken after 9, 11, and 14 h for phase I, II, and III, respectively. Cultivations were carried out in triplicates. The bar graphs represent the mean. The error bar indicates the standard deviation. CitZ, citrate synthase; MaeA, malic enzyme; MalS, malic enzyme; Mdh, malate dehydrogenase; MleA, malic enzyme; PdhABCD, pyruvate dehydrogenase; PckA, phosphoenolpyruvate carboxykinase; PPP, pentose phosphate pathway; PycA, pyruvate carboxylase; PykA, pyruvate kinase; YtsJ, malic enzyme (modified from Meyer and Stülke, 2013). 2OG, 2-oxoglutarate; DHAP, dihydroxyacetone phosphate; F6P, fructose-6-phosphate; FBP, fructose-bisphosphate; G6P, glucose-6-phosphate; GAP, D-glyceraldehyde 3-phosphate; R5P, ribose-5-phosphate; S7P, sedoheptulose-7-phosphate.

Investigating the growth behavior on xylose via the Weimberg pathway, a plateau indicating diauxic growth was observed for γ-PGA producing *B. subtilis* WB PX43-*pgs* and non-producing *B. subtilis* WB at optical densities of 5 and 2, respectively (Figure 4). To investigate this phenomenon, the metabolic state of the cells before (1), during (2), and after (3) the observed plateau was examined by metabolome analysis. The obtained metabolome data was analyzed by PCA. The changes in metabolite concentrations are presented by the score and loading plot (Figure 5) and by the metabolic profile shown in Figure 6. The PCA showed a separation of the three growth stages for the first component (PC1). Metabolites with high positive loading values are decreasing from the first to the third growth phase. Especially the concentrations of glycolysis and PPP intermediates decrease throughout the growth phases as indicated by high loading values for ribulose-5-phosphate, sedoheptulose-7-phosphate (S7P), FBP, and GAP. In contrast, glycolate and glycerate are increasing. These two metabolites are formed in several metabolic pathways such as the glyoxylate shunt or serine metabolism. Further, these two metabolites have been shown to be formed in the ethylene glycol degradation pathway (Antoniewicz, 2015). But none of these pathways is assigned to *B. subtilis*. Regarding the observed plateau, the metabolome data reveals that several metabolites from TCA cycle (succinate, fumarate, and malate) accumulate during the second phase that represents the lag phase (Figure 6). In contrast, pyruvate is clearly decreased during the plateau phase. This indicates that the bottleneck reaction hindering the growth in that phase seems to be the provision of pyruvate. Recently, labeling experiments with a switch from ^13^C-glucose to ^12^C-malate as carbon source demonstrated the inability of *B. subtilis* to instantaneously metabolize malate into the gluconeogenic direction (van Gulik et al., 2000). The delay in gluconeogenic malate metabolism is presumed to be due to the CcpN repression of the PEP carboxykinase (PckA). The regulation of the *B. subtilis* metabolism growing on xylose via the Weimberg pathway has to be further investigated.

### γ-PGA Production via Weimberg Pathway

**Figure 7:**
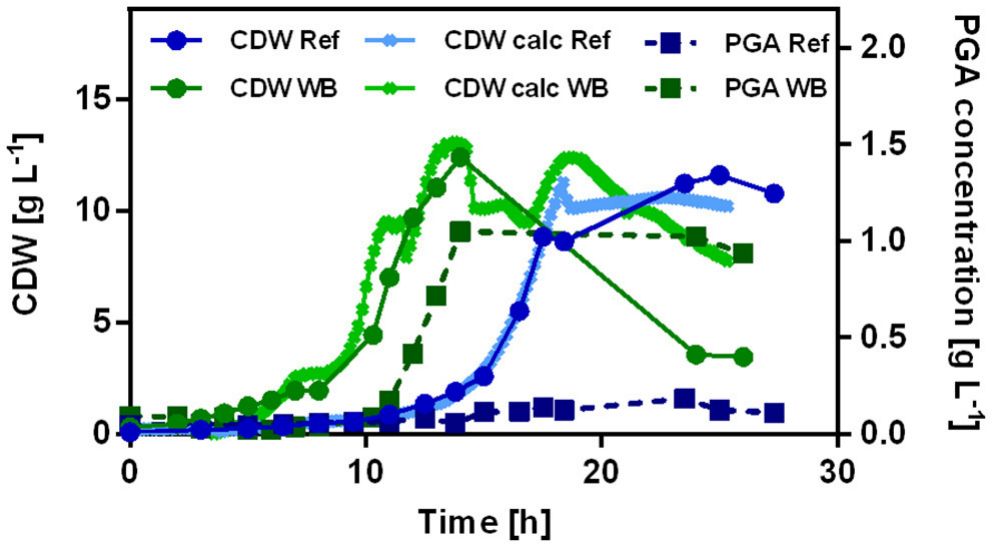
Batch fermentation of *B. subtilis* Ref PX43-*pgs* (blue) and *B. subtilis* WB PX43-*pgs* (green) on xylose. The cell dry weight was monitored offline and online using the BioR-CGQ (circles). The γ-PGA concentrations are depicted (squares).

To enable the decoupling of growth and production phase for mixed substrates, the combination of xylose-inducible and glucose-repressed promoter elements present in promoter PX43 was chosen. The decoupling of cell growth and γ-PGA production may be beneficial for a higher biocatalyst yield in the growth phase on glucose and an induced expression of the PGA synthetase after glucose is depleted. The promoter was integrated upstream of *pgs* in the strain background of the reference and recombinant Weimberg pathway strains. The strains were tested according to their growth behavior and γ-PGA production in batch fermentations with xylose as carbon source (Figure 7).

As in the shake flask experiments for the non-γ-PGA producing strains, the Weimberg pathway sustained with 0.37 h^−1^ a higher growth rate than observed for the reference strain (0.28 h^−1^). Consistently with the shake flask cultivations, the growth curve of *B. subtilis* WB PX43-*pgs* shows a plateau for ~1 h where the biomass concentration is not increasing. Further, *B. subtilis* WB PX43-pgs exhibits a shorter lag phase than the reference strain. The process time for *B. subtilis* WB PX43-*pgs* is shorter resulting in a higher γ-PGA production rate. For *B. subtilis* WB PX43-*pgs*, also the maximal obtained γ-PGA concentration was with 1.05 g L^−1^ higher than the 0.18 g L^−1^ γ-PGA for the reference. The obtained titer equals to yields of 0.06 and 0.01 C-mol γ-PGA per C-mol xylose for the recombinant Weimberg strain and the reference strain, respectively. Based on the results for the intracellular metabolite analysis, the higher γ-PGA production for the strain using the Weimberg pathway is likely caused by higher flux through the 2-oxoglutarate knot. Thereby a higher rate for the supply of glutamate is possible. However, the obtained yields are much lower than the theoretical yields for γ-PGA. To further investigate the theoretical flux distributions, the γ-PGA production was modeled using a stoichiometric model and FBA.

### Modeling of γ-PGA Production for Alternative Growth Conditions

The core metabolic model for γ-PGA-producing *B. subtilis* includes the central carbon metabolism, biomass formation, ATP maintenance, by-product formation and γ-PGA production. Based on the stoichiometric model, the theoretical C-mol yield for substrate mixtures containing xylose and glucose were calculated. For the calculation of γ-PGA production rates, a growth rate of 0.6 h^−1^ and an ATP maintenance coefficient of 9.9 mmol ${\text{g}}_{\text{cdw}}^{-1}$ h^−1^ were used as previously published for the genome-scale model of *B. subtilis* by Oh et al. (2007). Since the γ-PGA biosynthesis is an ATP-dependent reaction, the theoretical flux distribution is directly influenced by the chosen value for the ATP maintenance coefficient. The substrate uptake rate was fixed to 20 mmol ${\text{g}}_{\text{cdw}}^{-1}$ h^−1^, whereas the actual substrate uptake rate rates vary for the examined cultivation conditions. Based on these values, the γ-PGA production rate was calculated by FBA for different substrate compositions with the γ-PGA production rate as objective function. This approach enabled the theoretical calculation of optimal γ-PGA production rates as basis for targeted strain and process development.

**Figure 8:**
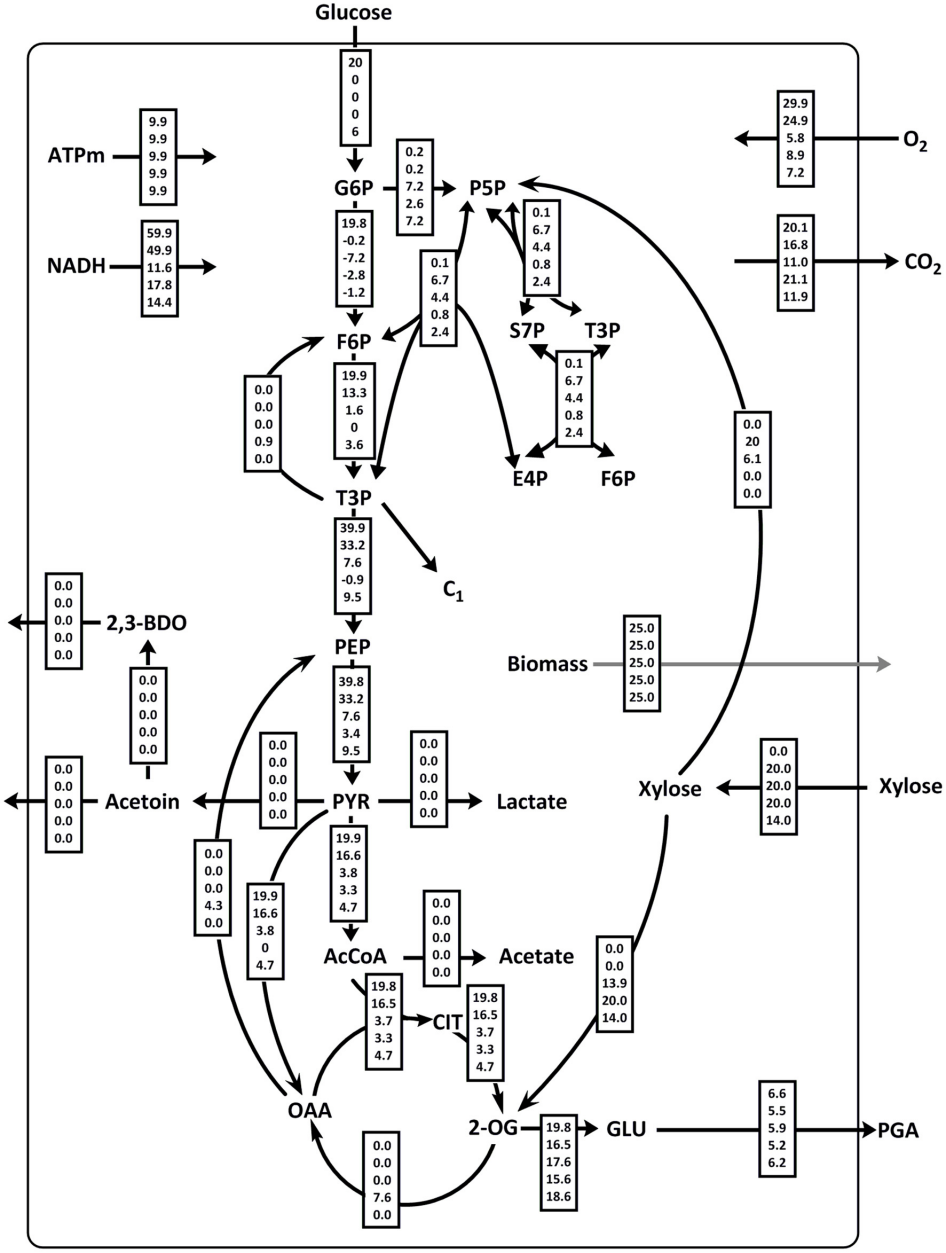
Theoretical flux distributions for γ-PGA producing *B. subtilis*. Flux balance analysis (FBA) was used to calculate the fluxes for growth on glucose (1), xylose via isomerase pathway (2), xylose via isomerase and Weimberg pathway (3), xylose via Weimberg pathway (4), and a glucose/xylose mixture (5).

The theoretical flux distributions for the optimization of γ-PGA synthesis rate under varying substrate conditions are shown in Figure 8. The growth on glucose as sole carbon source (1), xylose via the xylose isomerase pathway (2), using the Weimberg pathway (4), or both (3) and a calculated theoretical optimal mixture of glucose and xylose (5) were modeled. Glucose as carbon source results in the highest theoretical synthesis rate. Here, the substrate is mainly converted to glutamate employing glycolysis and the TCA cycle resulting in a theoretical glutamate yield of 99% of the substrate uptake. In this scenario, only 1% of the carbon flux is directed to the PPP at the glucose-6P branch point.

The metabolism of xylose as sole carbon source for growth and γ-PGA production was tested by two pathways, isomerase pathway and Weimberg pathway. When only the Weimberg pathway is present (4), xylose is completely converted to 2-oxoglutarate. For the supply of biomass precursors, 2-OG has to be partly converted to oxaloacetate that is subsequently used for gluconeogenesis via anaplerotic reactions (malic enzyme and PEP carboxykinase). For the given growth and ATP maintenance parameters, the theoretical flux distribution for the utilization of the PPP (2) is beneficial in terms of maximizing the γ-PGA production rate. The higher theoretical γ-PGA production rate for scenario (2) compared to (4) is likely due to precursor supply for biomass formation. Xylose metabolism via the Weimberg pathway requires energy-consuming gluconeogenesis. This catabolic pathway releases more carbon in form of CO_2_ since the Weimberg pathway includes the conversion 2-oxoglutarate to oxaloacetate which is further converted to phosphoenolpyruvate. In both reactions, CO_2_ is formed.

The theoretical γ-PGA production rate from xylose as sole carbon source is the highest when both xylose utilization pathways are present in the cells (3). In that case, ideally ~30% of xylose are converted to R5P using the isomerase pathway. The remaining xylose is metabolized via the Weimberg pathway resulting in a theoretical glutamate synthesis rate that is 88% of the substrate uptake rate. An even higher theoretical synthesis rate is achieved when a mixture of glucose and xylose is used (5). For this flux distribution, all xylose that is taken up is converted to glutamate via the Weimberg pathway. Precursors for biomass formation and redox equivalents are produced from glucose. Thereby a theoretical glutamate synthesis rate of 93% is calculated. While the synthesis rate is a bit lower as for glucose as carbon source, the theoretical carbon yield is higher for the substrate mix. However, the model does not include the consecutive uptake of the two substrates.

**Figure 9:**
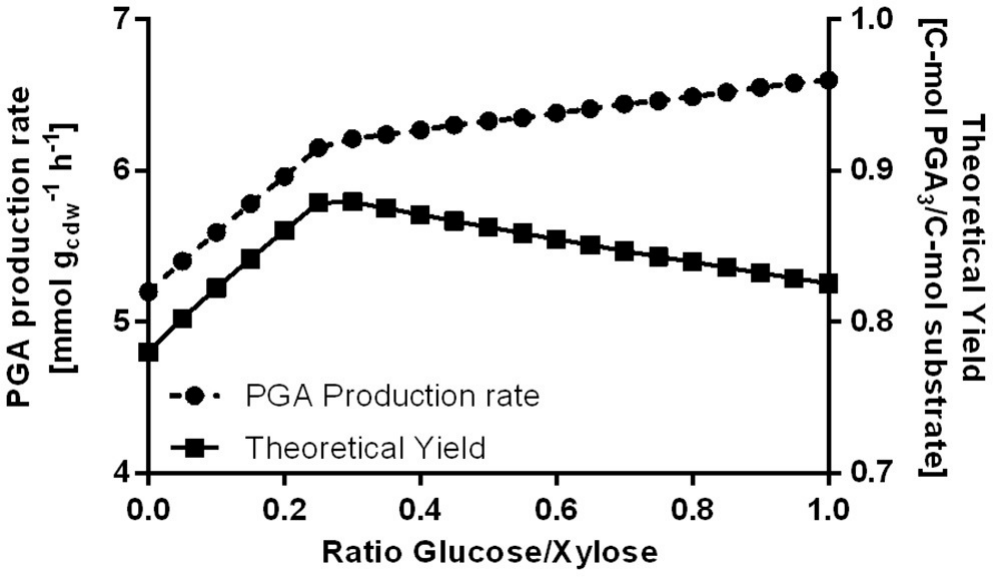
Modeling the γ-PGA production rate and the theoretical yield for varying substrate ratios. The total substrate uptake rate was kept constant at 20 mmol ${\text{g}}_{\text{cdw}}^{-1}$ h^−1^. The biomass formation rate was set to 25 mmol ${\text{g}}_{\text{cdw}}^{-1}$ h^−1^ and the ATP maintenance coefficient was 9.9 mmol ${\text{g}}_{\text{cdw}}^{-1}$ h^−1^.

Modeling the flux distributions for γ-PGA-producing *B. subtilis*, the model revealed that the Weimberg pathway for xylose utilization is superior to the native xylose isomerase pathway when mixtures of glucose and xylose are used as substrate. Based on the given metabolic model, the optimized γ-PGA production rate and the theoretical yield were calculated as a function of substrate composition by FBA. The substrate was a mixture of glucose and xylose. The variation in the portions of glucose and xylose was modeled (Figure 9). When using a fixed growth rate, ATP maintenance rate and overall substrate uptake rate, the γ-PGA production rate increases with an increase in the glucose portion. However, the theoretical C-mol yield exhibits an optimum for a substrate mixture with 30% glucose. The absolute value for the theoretical yield varies between 0.78 and 0.88 C-mol γ-PGA/C-mol substrate. While the formation of biomass and ATP is more efficient using glucose via glycolysis and TCA as substrate, the availability of xylose via the Weimberg pathway is beneficial for maximizing the theoretical γ-PGA yield since xylose is converted to glutamate without carbon loss in form of CO_2_.

### γ-PGA Production Under Varying Substrate Concentrations

**Figure 10:**
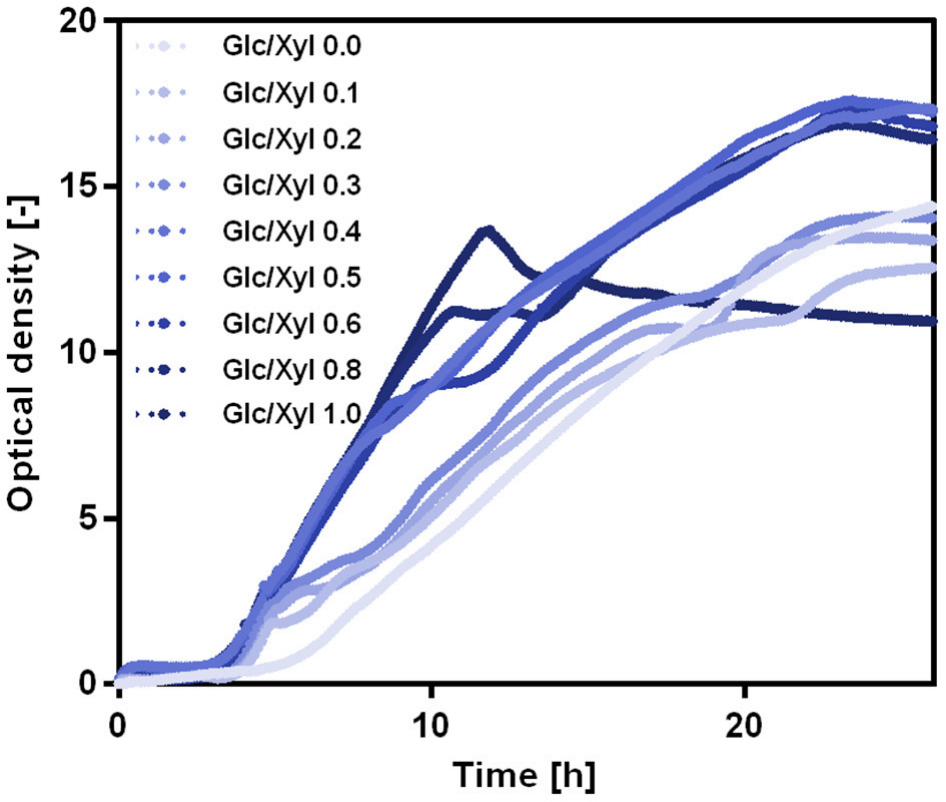
Growth curves for *B. subtilis* WB PX43-*pgs* with varying substrate ratios. The total substrate of 20 g L^−1^ was varied from 0 to 100% glucose (light blue to dark blue). The cultivations were carried out in 500 mL shake flasks with 50 mL filling volume and 200 rpm shaking frequency. The growth curves were monitored online using the CGQ (Aquila Biolabs). The data represent the mean of triplicate cultivations.

For growth of *B. subtilis* WB PX43-pgs on a substrate mix of glucose and xylose, glucose is the preferred substrate. The growth curves on a mixture of glucose and xylose demonstrate a short lag phase indicating the shift from glucose to xylose metabolism that occurs later the higher the portion of glucose was chosen (Figure 10). Moreover, the growth curves emphasize that the growth rate of *B. subtilis* WB PX43-pgs on glucose is higher than on xylose (~0.6 and 0.4 h^−1^, respectively).

**Figure 11:**
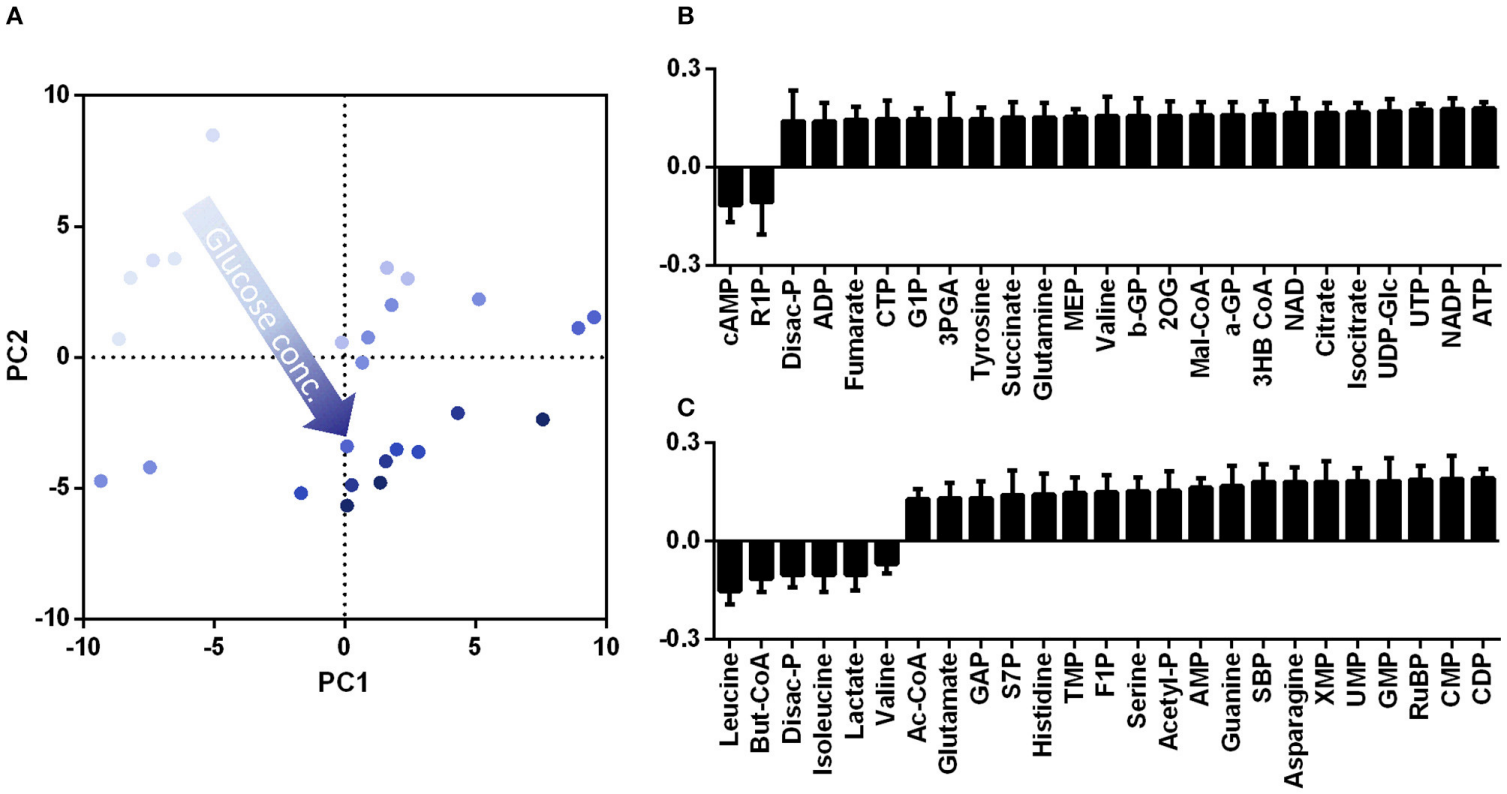
Principal component analysis for substrate variations in *B. subtilis* WB PX43-*pgs* cultivations. The score plot **(A)** and loading data for PC1 **(B)** and PC2 **(C)** are given. The total substrate of 20 g L^−1^ was varied from 0 to 100% glucose (light blue to dark blue). The loading data for PC1 and PC2 is given for the metabolites with the highest impact on the principal components.

The effect of substrate ratio on the metabolism was also investigated by metabolome analysis. Intracellular metabolite extractions were carried out after 9 h at optical densities ranging between 8 for higher glucose concentrations and 6 for lower glucose concentrations. Differences in the intracellular metabolites caused by the substrate variation were evaluated by PCA (Figure 11). The first component is mainly representing the energy status of the cells, showing increased values for ATP, NADP, and NAD. The cAMP concentration decreased with PC1. The presence of cAMP in *B. subtilis* was detected for oxygen limited conditions (Mach et al., 1984). However, the role of cAMP is not comparable to the role in other bacteria such as *E. coli* where it is involved in altered gene expression as a second messenger controlling substrate uptake, motility or virulence (Makman and Sunderland, 1965; de Crombrugghe et al., 1984; Kalia et al., 2013). Besides energy-metabolism related metabolites, several TCA cycle intermediates (citrate, 2-OG, and succinate) are increased for increasing PC1 values. These findings agree with the results for the comparison of the Weimberg pathway and the xylose isomerase pathway. As stated before, the conversion of xylose via the Weimberg pathway likely causes a higher TCA cycle flux. As a result, the concentrations of TCA cycle intermediates decrease with higher xylose concentrations. The loading data for PC2 indicate increasing lactate concentrations for higher glucose concentration likely caused by induced overflow metabolism (Nakano et al., 1997; Cruz Ramos et al., 2000; Sonenshein, 2007). Moreover, some amino acids that are derived from glycolysis intermediates (leucine, valine) exhibit higher concentrations for higher glucose ratios. With higher xylose ratios several intermediates of purine metabolism correlate positively. Furthermore, some PPP metabolites such as RuBP, SBP, S7P, and the therefrom derived amino acid histidine exhibit increasing concentrations. Higher concentrations for intermediates from PPP have also been found when comparing the Weimberg pathway to the xylose isomerase pathway.

**Figure 12:**
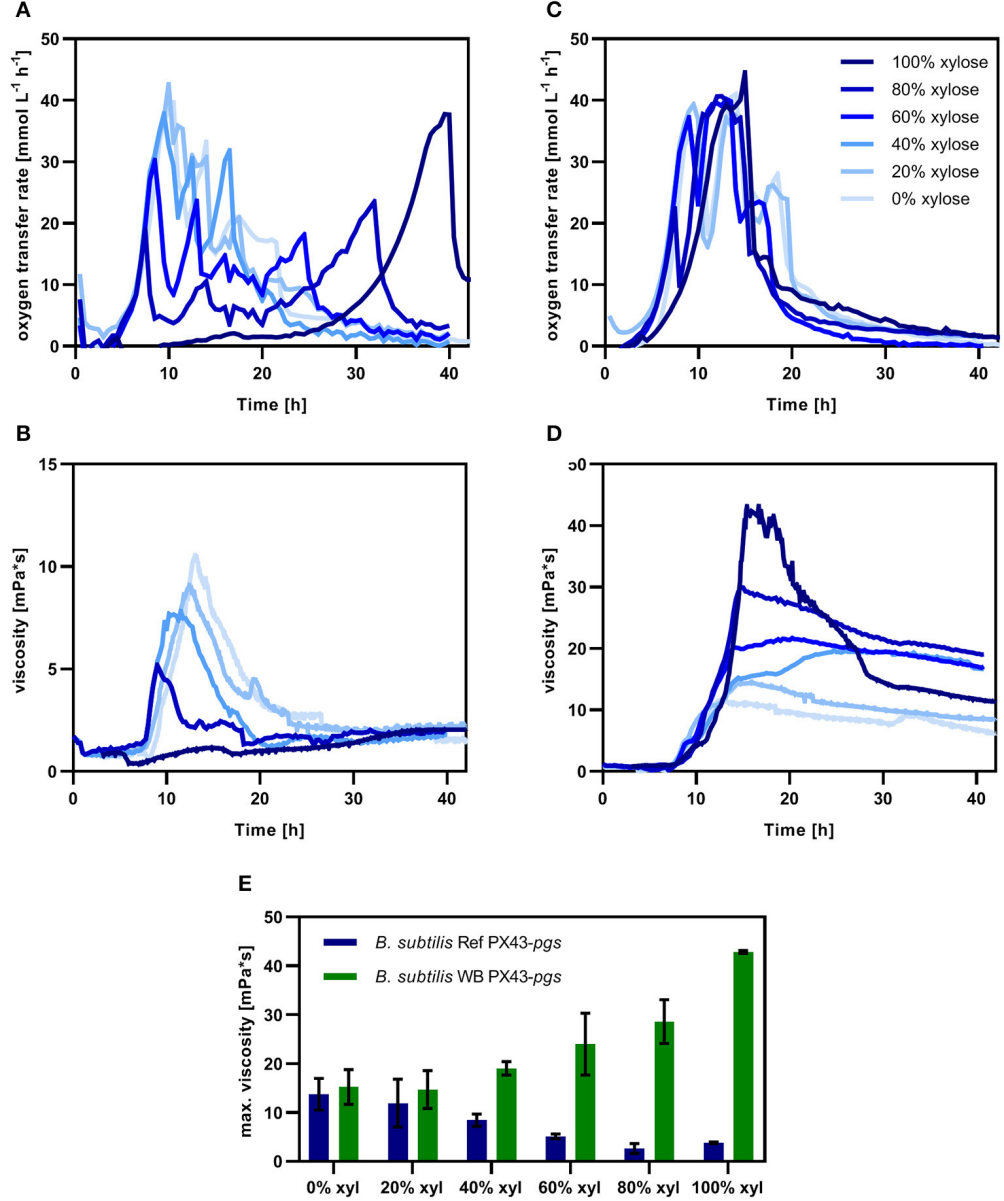
Growth and γ-PGA production of engineered *B. subtilis* Ref PX43-*pgs* (reference; **A,B**) and *B. subtilis* WB PX43-*pgs* **(C,D)** in glucose/xylose minimal medium in shake flask cultivations. The oxygen transfer rate (OTR) representing the cell growth and metabolic activity of the cultured microorganisms is shown in **(A)** for the reference strain and in **(C)** for the Weimberg mutant. The γ-PGA measured as online viscosity is shown in **(B)** for *B. subtilis* Ref PX43-*pgs* and in **(D)** for *B. subtilis* WB PX43-*pgs* with glucose/xylose ratios varying from 0% xyl (100% glucose, 0% xylose) to 100% xyl (0% glucose, 100% xylose). The total substrate concentration was kept constant at 20 g L^−1^ corresponding to 0.67 C-mol L^−1^. The cultivations were carried out in triplicates for 20, 40, 60, and 80% xylose and in duplicates for 0 and 100% xylose. The obtained maximal viscosity for the reference (blue) and Weimberg mutant (green) is given in **(E)**.

The γ-PGA production of *B. subtilis* Ref PX43-pgs (Figures 12A,B) and *B. subtilis* WB PX43-pgs (Figures 12C,D) was investigated for several substrate ratios including 0, 20, 40, 60, 80, and 100% xylose. The γ-PGA production was monitored by on-line viscosity measurements (Sieben et al., 2019) and off-line γ-PGA concentration determination using the CTAB assay. Growth was closely monitored by measuring the OTR. Regestein et al. have shown that the γ-PGA concentration is closely related to viscosity (Regestein Née Meissner et al., 2017).

The OTR increases simultaneously during the first 7.5 h for both strains and all substrate ratios except for 100% xylose (Figure 12A for reference and C for Weimberg mutant). Since glucose is the preferred substrate and the metabolic conversion of glucose is the same for both strains, no differences are observed until this time point. After that, the OTR drops for 80% xylose for both strains. For this glucose ratio, *B. subtilis* Ref PX43-*pgs* grows for 34 h. After 34 h, the OTR drops below 5 mmol L^−1^ h^−1^, which is slower compared to the Weimberg mutant. These results are in concordance with the lower growth rate of *B. subtilis* Ref PX43-*pgs* on xylose (Table 2 and Figure 7). Independent of the substrate composition, three maxima are observed for the OTR of *B. subtilis* Ref PX43-*pgs* (Figure 12A). These maxima correspond to the growth on glucose, on by-products such as 2,3-butanediol, and on xylose. For 20% xylose, a plateau at 40 mmol L^−1^ h^−1^ indicates an oxygen limitation. The determination of γ-PGA production by online viscosity measurements shows an increase of viscosity from 7 h on for all substrate compositions except 100% xylose (reference) (Figure 12B). The maximal viscosity strongly depends on the substrate composition reaching a higher viscosity the more glucose is present. The highest viscosity obtained for *B. subtilis* Ref PX43-*pgs* was 14 mPa s for 100% glucose. This viscosity corresponds to 4.7 g L^−1^ γ-PGA as determined by CTAB assay. The OTR curve for *B. subtilis* Ref PX43-*pgs* on 100% xylose increases only after 10 h indicating a long lag phase for growth on xylose via the isomerase pathway. γ-PGA was poorly observed for the reference strain under these conditions.

For *B. subtilis* WB PX43-*pgs*, the OTR increased simultaneously to 22 mmol L^−1^ h^−1^ for all substrate ratios except 100% xylose during the first 7.5 h (Figure 12C). At this time point, the OTR for the cultures with the lowest glucose portion drops to 10 mmol L^−1^ h^−1^, indicating the depletion of glucose. This decline in OTR was observed for the other cultures after 8.5, 9, and 9.4 h for 40, 60, and 80% glucose, respectively. In all cultures, the OTR starts increasing again approximately 0.5 h after the drop indicating the shift from glucose metabolism to xylose metabolism. For glucose ratios of 0.2, 0.4, and 0.6, the OTR is constant at 40 mmol L^−1^ h^−1^ between 10.5 and 13.5 h. The constant OTR indicates an oxygen limitation. After 13.5 h, the OTR started to decline and reached ~0 mmol L^−1^ h^−1^ after 35 h. For the two highest glucose portions, a second OTR maximum after 13 and 14.5 h for 100 and 80% glucose, respectively, is detected, indicating the growth on a previously produced overflow metabolite. After that maximum, the OTR is constant at 25 mmol L^−1^ h^−1^ between 15.5 and 24 h. After 24 h, the OTR drops and reaches 0 mmol L^−1^ h^−1^ after 35 h. In accordance with the lower growth rate of *B. subtilis* WB PX43-*pgs* on xylose, the OTR increases slower for 100% xylose reaching the maximum after 15 h.

The γ-PGA production of *B. subtilis* WB PX43-*pgs* was monitored by online viscosity measurement (Figure 12D). The highest viscosity of 43 mPa·s was observed for the culture with xylose as sole carbon source. With increasing glucose ratios, the maximal viscosity decreased to 30, 22, 19, and 19 mPa·s for 20, 40, 60, and 80% glucose, respectively. After substrate depletion, the viscosity slightly decreased indicating the degradation of the produced γ-PGA. Besides the online viscosity measurement, the γ-PGA concentration was determined using the CTAB assay, with the same result: the maximal γ-PGA concentration of 5 g L^−1^ was obtained with 80% xylose. The produced γ-PGA corresponds to a C-mol yield of 0.26. With decreasing xylose portion, the γ-PGA titer decreased gradually with 4.5, 4, and 3.5 g L^−1^ for 60, 40, and 20% xylose, respectively.

In contrast to the Weimberg mutant, the γ-PGA production for *B. subtilis* Ref PX43-pgs was higher with higher glucose ratios (Figure 12E). Moreover, also the maximal viscosity was increased from 19 mPa·s for the reference to 30 mPa·s for the Weimberg mutant. Therefore, for the production of γ-PGA, the utilization of the Weimberg pathway is clearly superior to the xylose isomerase pathway.

## Discussion

Integration of the *C. crescentus xylXABCD* genes was demonstrated to enable bacteria such as *Pseudomonas* (Meijnen et al., 2009) and *Corynebacterium glutamicum* (Radek et al., 2014) to grow on xylose as sole carbon and energy source. The direct conversion of xylose to the C5-compound 2-oxoglutarate without carbon loss is beneficial for γ-PGA production as well as for amino acids derived from 2-oxoglutarate. However, the reported maximal growth rate with μ = 0.21 h^−1^ for *P. putida* S12 (Meijnen et al., 2009) and μ = 0.07 h^−1^ for *C. glutamicum* (Radek et al., 2014) were low. In both cases, plasmid-based expression of the enzymes of the Weimberg pathway was used. The genomic integration of *xylXABCD* in *B. subtilis* WB led to a growth rate of 0.43 h^−1^ (Table 2). The genomic integration circumvents the metabolic burden of plasmid replication. The replacement of the native *B. subtilis* xylose operon allowed for the regulation of expression by the native *B. subtilis* xylose promoter. The resulting growth rate was even higher than for the wild-type *B. subtilis* using the isomerase pathway.

In *P. putida* and *C. glutamicum*, the accumulation of xylonate was observed in the supernatant. Xylonate accumulation strongly indicates that the xylonate dehydratase reaction converting xylonate to 2-keto-3-desoxy-xylonate is the rate-limiting step of the pathway. The inefficient conversion of xylonate is likely one factor for the reduced growth rates in these bacteria. For *B. subtilis* WB, the higher growth rate indicates a sufficient activity of all enzymes. Nevertheless, a bi-phasic (diauxic) growth with a lag phase between the two growth phases was observed. Here, intracellular metabolite measurements revealed the accumulation of TCA intermediates such as malate and fumarate. This indicates that the conversion of malate and oxaloacetate to pyruvate and phosphoenolpyruvate are rate-limiting steps in *B. subtilis* WB.

The two xylose utilization pathways, xylose isomerase and Weimberg pathway were compared with regard to the precursor supply for γ-PGA production. In contrast to the hypothesized higher 2-oxoglutarate supply for the Weimberg mutant, the concentration of most TCA cycle intermediates was higher for the xylose isomerase pathway. The metabolite measurements indicate a higher activity TCA cycle when xylose is converted to 2-oxoglutarate. This results in lower metabolite concentrations for TCA cycle intermediates. These results are consistent with the metabolome analysis for γ-PGA-producing *B. licheniformis* (Mitsunaga et al., 2016). For higher fluxes toward γ-PGA when glycerol is used as carbon source, the precursor concentrations of citrate, isocitrate, and 2-oxoglutarate are significantly lower than for glucose with a lower γ-PGA production rate. The higher TCA cycle activity was demonstrated to result in higher γ-PGA synthesis for the Weimberg mutant.

Several studies demonstrated an efficient γ-PGA synthesis with glutamic acid as additional carbon source (Cromwick et al., 1996; Richard and Margaritis, 2003). The majority of these studies involve wild-type γ-PGA producers that require glutamate for γ-PGA synthesis. For glutamate-independent production of γ-PGA the media commonly contain citric acid to enable high γ-PGA titers (Kongklom et al., 2017). In all cases, the γ-PGA production is greatly increased when direct glutamate precursors are used as substrates. The direct conversion of xylose to 2-oxoglutarate implemented in this study presents an alternative solution for higher precursor supply from cheaper substrates. Since up to 25% of lignocellulosic biomass are made up from pentoses (Lee, 1997), their use is essential to efficiently produces bio-based products.

When glucose and L-glutamate were used as carbon sources, only 6–9% of the glutamate that was incorporated into γ-PGA was *de novo* synthesized from glucose (Yao et al., 2010). Therefore, an increase in the initial glucose concentration mainly resulted in higher biomass formation instead of higher γ-PGA synthesis. Hence, the utilization of two carbon sources may be beneficial if one carbon source is used for growth and energy production and another one is used for γ-PGA precursor supply. The investigation of the theoretical flux distributions emphasized this hypothesis. A higher theoretical γ-PGA production rate was observed for glucose and xylose mixtures compared to xylose alone. For substrate mixtures, glucose was converted to biomass precursors and used as energy source. Xylose was converted to glutamate without carbon loss. However, the consecutive uptake of the two substrates is not considered in the theoretical flux distribution. Further engineering may focus on the utilization of both carbon sources (Wu et al., 2016). In this study, the integration of promoter PX43 for PGA synthetase aimed at the discrimination of growth phase on glucose and production phase on xylose. Since the viscosity started increasing before glucose depletion, the PGA synthetase was expressed despite of the cre site for glucose repression. Furthermore, the theoretical flux distributions strongly depend on the growth rate and substrate uptake rate. A growth rate of 0.6 h^−1^ as used for the FBA was only proven to be true for growth on glucose. The changing growth rate throughout the cultivation was not considered.

In this study, the Weimberg pathway was successfully implemented into *B. subtilis* to achieve high γ-PGA production from biomass-derived substrates. Thereby, the carbon loss for utilization of xylose as substrate was minimized. The use of a mixture of glucose and xylose enabled the γ-PGA synthesis with a yield of 0.26 C-mol/C-mol. The yield may further be improved by strain and process development to achieve resource efficient γ-PGA production from biomass-derived substrates.

## Data Availability Statement

All datasets generated for this study are included in the article/Supplementary Material.

## Author Contributions

BH and LB conceived and designed the study. SP and EF contributed to the design and data analysis of metabolomics experiments. BH, KH, RH, LB, and JB contributed to the design and data analysis of online viscosity experiments. KH and RH performed the online viscosity measurements. BH performed the remaining experiments and drafted the manuscript. All authors revised the manuscript and approved the final manuscript.

### Conflict of Interest

The authors declare that the research was conducted in the absence of any commercial or financial relationships that could be construed as a potential conflict of interest.
