# Supplementary material for: Comparison of Isomerase and Weimberg Pathway for γ-PGA Production From Xylose by Engineered Bacillus subtilis
Source: Front Bioeng Biotechnol. 2020 Jan 21;7:476. doi: 10.3389/fbioe.2019.00476 (PMC6985040; doi:10.3389/fbioe.2019.00476)
Supplement: Supplementary file 1 [file Table_1.DOCX]

Supplementary Material

# Supplementary table

Supplementary Table 1: Primers used in this study

| **name** | **Sequence (5’-3’)** | **Purpose** |
| --- | --- | --- |
| BS-25 | GGGCGTGTCAATAATATCACTC | Vector backbone linearization |
| BS-26 | GGGCGACGGATGGTGATCCC | Vector backbone linearization |

| BS-085 | GGGATCACCATCCGTCGCCCGATTGTTGATAATAACACCG | Amplification of TS1 for pBs-17 |
| --- | --- | --- |
| BS-086 | ATCTCCAGACAGATTCGCTATGTACTATGTTG | Amplification of TS1 for pBs-17 |
| BS-087 | TAGCGAATCTGTCTGGAGATCAACCGAAG | Amplification of TS2 for pBs-17 |
| BS-088 | GTGATATTATTGACACGCCCGTAAGCTACAGCTGGAATTTG | Amplification of TS2 for pBs-17 |
| BS-092 | GGGAGCTGGCGCAGTCTGGAGATCAACCGAAG | Amplification of TS2 for pBs-18 |
| BS-098 | GCGACGAGGTGATTTC | Sequencing of pBs-18 integration |
| BS-099 | GGGATCACCATCCGTCGCCCGCTGATCGTAGATGAAGATG | Amplification of TS1 for pBs-19 |
| BS-100 | CAATGATACACGATCGCCGGAACACAAAAAG | Amplification of TS1 for pBs-19 |
| BS-101 | TGTGTTCCGGCGATCGTGTATCATTGGTTTAC | Amplification of P43 for pBs-19 |
| BS-102 | CCAATAATAAATCCATGTGTACATTCCTCTCTTAC | Amplification of P43 for pBs-19 |
| BS-103 | GAGGAATGTACACATGGATTTATTATTGGCTCTTCTCCC | Amplification of TS2 for pBs-19 |
| BS-104 | ACAGAGTGATATTATTGACACGCCCACCGCCGAGCGTAGAAATG | Amplification of TS2 for pBs-19 |
| BS-155 | GTCTACAAACCACGTGATTTCCCCCTTAAAAATAAATTC | Amplification of TS1 for pBs-24 |
| BS-156 | AGGGGGAAATCACGTGGTTTGTAGACGTCTTTTAG | Amplification of *xylXA* for pBs-24 |
| BS-157 | ATCCCGGCCGTAGTTATGACCATGAATAAGAAGTC | Amplification of *xylXA* for pBs-24 |
| BS-158 | TTCATGGTCATAACTACGGCCGGGATGAAGTG | Amplification of *xylCD* for pBs-24 |
| BS-159 | TGATCTCCAGACTGCGCCAGCTCCCTCAATGATTATGCCGAGGCAG | Amplification of *xylCD* for pBs-24 |
| BS-224 | GTGTACATTCCTCTCTTAC | Amplification of backbone for pBs-34 |
| BS-225 | GGGCGTGTCAATAATATC | Amplification of backbone for pBs-34 |
| BS-226 | GGTAAGAGAGGAATGTACACATGTCCTCAGCAATTTACCCAAG | Amplification of *xylB* for pBs-34 |
| BS-227 | TCTGGGTCGCTCAACGCCATCCCGCATC | Amplification of *xylB* for pBs-34 |
| BS-228 | ATGGCGTTGAGCGACCCAGACATGACATC | Amplification of TS2 for pBs-34 |
| BS-229 | GTGATATTATTGACACGCCCAGGCGTCTTCCAAGATCAAAAC | Amplification of TS2 for pBs-34 |

Supplementary Table 2: Metabolite concentrations

|  | ***B. subtilis* Ref** | | ***B. subtilis* Ref PX43-*pgs*** | | ***B. subtilis* WB** | | ***B. subtilis* WB PX43-*pgs*** | |
| --- | --- | --- | --- | --- | --- | --- | --- | --- |
| **Metabolite** | **Mean** | **Std** | **Mean** | **Std** | **Mean** | **Std** | **Mean** | **Std** |
| 2-Isopropylmalate | 3,3951 | 0,7030 | 5,2630 | 1,1180 | 3,3656 | 0,2625 | 3,4382 | 0,7280 |
| 2OG | 2,8172 | 0,1232 | 1,4388 | 0,1863 | 1,4536 | 0,2747 | 1,5636 | 0,1572 |
| 3HB CoA | 0,1665 | 0,0959 | 0,1397 | 0,0378 | 0,1763 | 0,0256 | 0,3582 | 0,1134 |
| 3PGA | 3,8567 | 0,3670 | 6,4028 | 0,9027 | 7,1000 | 0,0914 | 4,6020 | 0,4716 |
| 6PGA | 0,5333 | 0,0388 | 0,5786 | 0,1032 | 3,7568 | 0,8176 | 1,1741 | 0,1787 |
| Acetyl CoA | 0,0307 | 0,0073 | 0,1633 | 0,0512 | 0,5813 | 0,2060 | 0,0894 | 0,0161 |
| Acetyl-P | 0,0760 | 0,0517 | 0,0706 | 0,0741 | 0,3769 | 0,1106 | 0,2098 | 0,0355 |
| Adenine | 0,0217 | 0,0037 | 0,0783 | 0,0230 | 0,4507 | 0,0687 | 0,1836 | 0,0116 |
| Adenosine | 0,0019 | 0,0008 | 0,0053 | 0,0015 | 0,0376 | 0,0115 | 0,0034 | 0,0011 |
| ADP | 1,0586 | 0,0327 | 0,7347 | 0,1165 | 0,2904 | 0,0630 | 0,7750 | 0,0842 |
| a-GP | 2,7670 | 0,1539 | 6,6980 | 0,6016 | 5,4079 | 1,1017 | 2,3910 | 0,4065 |
| AICAR | 0,0370 | 0,0138 | 0,0465 | 0,0139 | 0,0878 | 0,0089 | 0,0494 | 0,0072 |
| AMP | 9,1520 | 1,8232 | 6,1049 | 0,5463 | 2,4150 | 0,4227 | 3,9871 | 1,0283 |
| Arginine | 0,0098 | 0,0021 | 0,0078 | 0,0030 | 0,0587 | 0,0132 | 0,0062 | 0,0022 |
| Asparagine | n.d. | 0,0000 | 0,0091 | 0,0086 | 0,1163 | 0,0130 | 0,1442 | 0,0211 |
| Aspartate | 0,4588 | 0,0723 | 2,8593 | 0,0691 | -0,0181 | 0,0000 | 3,0284 | 0,7749 |
| ATP | 0,9739 | 0,2651 | 0,8008 | 0,0781 | 0,3155 | 0,1031 | 2,3470 | 0,4114 |
| b-GP | 6,2488 | 0,2257 | 15,6938 | 2,3237 | 11,2040 | 2,3262 | 5,2562 | 0,9975 |
| BPG | 0,2740 | 0,0250 | 0,3824 | 0,0758 | 0,5280 | 0,1048 | 0,6714 | 0,0327 |
| Butyryl CoA | 0,0030 | 0,0017 | 0,0018 | 0,0010 | 0,0020 | 0,0012 | 0,0022 | 0,0007 |
| cAMP | n.d. | 0,0000 | 0,0006 | 0,0008 | 0,1100 | 0,0159 | 0,0331 | 0,0100 |
| CDP | 0,7563 | 0,0481 | 0,3956 | 0,0597 | 0,3437 | 0,0736 | 0,5812 | 0,1042 |
| Citrate | 3,5531 | 0,3397 | 5,4853 | 0,5621 | 6,6567 | 0,4491 | 17,5210 | 1,0292 |
| CMP | 3,2650 | 0,9461 | 3,4746 | 0,6124 | 2,3952 | 0,2961 | 1,4509 | 0,1671 |
| CoA | 0,1604 | 0,0318 | 0,1245 | 0,0097 | 0,8003 | 0,2311 | 0,7155 | 0,2723 |
| CTP | 0,5729 | 0,0798 | 0,2284 | 0,0291 | 0,1853 | 0,0473 | 1,0191 | 0,1733 |
| Cysteine | 0,0091 | 0,0019 | 0,0156 | 0,0058 | 0,0109 | 0,0050 | 0,0064 | 0,0014 |
| Cytidine | 0,1214 | 0,0248 | 0,5406 | 0,0519 | 1,9939 | 0,4530 | 0,0937 | 0,0374 |
| DHAP | 0,1202 | 0,0317 | 1,8264 | 0,7831 | 6,2059 | 2,1245 | 1,4213 | 0,3372 |
| Disaccharide-P | 0,0114 | 0,0022 | 0,0055 | 0,0032 | 0,1381 | 0,0211 | 0,0956 | 0,0175 |
| F1P | 0,8574 | 0,1781 | 1,2854 | 0,1523 | 12,5491 | 1,7105 | 1,1570 | 0,0759 |
| F6P | 0,5520 | 0,0416 | 0,4119 | 0,1032 | 3,4992 | 1,4111 | 0,2318 | 0,0316 |
| FBP | 1,5517 | 0,4584 | 7,1163 | 1,0712 | 22,5670 | 2,1618 | 12,1592 | 1,4360 |
| Fumarate | 0,0211 | 0,0087 | 0,0432 | 0,0119 | 0,1049 | 0,0017 | 0,0866 | 0,0116 |
| G1P | 0,5522 | 0,0884 | 0,4940 | 0,0645 | 1,4771 | 0,3311 | 0,2036 | 0,0267 |
| G6P | 0,1017 | 0,0304 | 0,4600 | 0,1191 | 3,6780 | 1,1943 | 0,2649 | 0,0398 |
| GAP | 0,2919 | 0,0393 | 0,4103 | 0,1003 | 1,2682 | 0,1598 | 0,4680 | 0,1479 |
| GDP | 0,3708 | 0,0320 | 0,2999 | 0,0703 | 0,1703 | 0,0339 | 0,1232 | 0,0184 |
| Glutamate | 9,8169 | 0,7395 | 13,1797 | 0,9662 | 11,9496 | 0,8199 | 8,7284 | 1,0740 |
| Glutamine | 6,9439 | 0,5743 | 1,9119 | 0,1657 | 0,9917 | 0,1501 | 0,2459 | 0,0888 |
| Glycerate | 0,1726 | 0,0083 | 0,0910 | 0,0274 | 0,1278 | 0,0284 | 0,1879 | 0,0598 |
| Glycolate | 0,8982 | 0,0755 | 0,1556 | 0,0859 | 0,2976 | 0,0683 | 1,1544 | 0,2193 |
| GMP | 1,7247 | 0,2820 | 1,3357 | 0,3635 | 0,8476 | 0,1376 | 1,3539 | 0,1687 |
| GTP | 0,4454 | 0,0413 | 0,3686 | 0,0325 | 0,3779 | 0,0260 | 0,7138 | 0,0968 |
| Guanine | 0,0387 | 0,0156 | 0,0048 | 0,0008 | 0,1057 | 0,0263 | 0,0496 | 0,0088 |
| Guanosine | 0,0481 | 0,0144 | 0,0712 | 0,0198 | 0,4048 | 0,1034 | 0,0403 | 0,0160 |
| Histidine | 0,0100 | 0,0007 | 0,0143 | 0,0026 | 0,0189 | 0,0004 | 0,0126 | 0,0034 |
| IMP | 0,0759 | 0,0046 | 0,1117 | 0,0144 | 0,3922 | 0,1362 | 0,7773 | 0,2122 |
| Isocitrate | 3,6367 | 0,2974 | 5,1954 | 0,6124 | 6,9124 | 0,8566 | 17,9574 | 1,3742 |
| Isoleucine | 0,0650 | 0,0281 | 0,0701 | 0,0292 | 0,4324 | 0,1269 | 0,1380 | 0,0635 |
| KDPG | 0,0157 | 0,0017 | 0,0534 | 0,0120 | 0,0035 | 0,0056 | n.d. | 0 |
| Lactate | n.d. | 0,0000 | 0,5585 | 0,2985 | 0,2730 | 0,0515 | 0,4537 | 0,0438 |
| Leucine | 0,0281 | 0,0127 | 0,0122 | 0,0063 | 0,0211 | 0,0093 | 0,0162 | 0,0097 |
| Lysine | 0,3884 | 0,0393 | 0,1149 | 0,0123 | 0,0711 | 0,0111 | 0,0136 | 0,0061 |
| Malate | 0,0000 | 0,0082 | 0,0092 | 0,0149 | 0,3305 | 0,0346 | 0,2440 | 0,0425 |
| Malonyl CoA | 0,1261 | 0,0697 | 0,1131 | 0,0370 | 0,1537 | 0,0207 | 0,2737 | 0,0981 |
| MEP | 1,2084 | 0,1976 | 0,7051 | 0,0835 | 0,2966 | 0,0573 | 0,0628 | 0,0052 |
| Methionine | 0,1660 | 0,0317 | 0,1132 | 0,0136 | 0,2480 | 0,0415 | 0,0977 | 0,0206 |
| NAD | 4,9844 | 0,4939 | 3,6754 | 0,7800 | 3,2384 | 0,3681 | 4,2479 | 0,5290 |
| NADP | 3,8972 | 0,5315 | 3,2896 | 0,7126 | 2,5604 | 0,1954 | 1,8408 | 0,1883 |
| Nicotinate | 0,2745 | 0,0290 | 1,9854 | 0,6536 | 0,7372 | 0,2323 | 1,2073 | 0,3582 |
| Orotate | 0,1683 | 0,0128 | 0,9307 | 0,2682 | 1,7751 | 0,4504 | 7,5028 | 2,5555 |
| Pantothenate | 0,8753 | 0,1871 | 1,5362 | 0,0621 | 0,8116 | 0,1113 | 1,7132 | 0,4706 |
| PEP | 1,0335 | 0,1188 | 2,6422 | 0,4033 | 1,8938 | 0,1008 | 1,5383 | 0,2312 |
| Phenylalanine | 0,1494 | 0,1308 | 0,0368 | 0,0032 | 0,0954 | 0,0231 | 0,0806 | 0,0289 |
| PQQ | 1,0056 | 0,2233 | 0,7191 | 0,3481 | 0,9725 | 0,2460 | 0,8416 | 0,3015 |
| Proline | 0,0173 | 0,0069 | 0,0143 | 0,0051 | 0,2087 | 0,0640 | 0,0989 | 0,0184 |
| PRPP | 0,1212 | 0,0124 | 0,1419 | 0,0635 | 0,3030 | 0,0988 | 0,6768 | 0,1377 |
| Pyroglutamate | 0,0298 | 0,0085 | 0,1079 | 0,0547 | 0,0604 | 0,0229 | 0,0085 | 0,0293 |
| Pyruvate | 0,4484 | 0,0512 | 0,4169 | 0,1255 | 0,3775 | 0,0794 | 0,5143 | 0,1211 |
| R1P | 0,4786 | 0,0934 | 1,9021 | 0,4556 | 11,5654 | 0,5014 | 7,9701 | 0,3865 |
| R5P | 0,6151 | 0,0346 | 1,5876 | 0,4669 | 3,2955 | 0,9449 | 1,1122 | 0,3396 |
| Ru5P | 0,8193 | 0,1399 | 3,2394 | 1,0722 | 9,9709 | 5,0675 | 1,6439 | 0,3762 |
| RuBP | 0,0098 | 0,0041 | 0,1000 | 0,0332 | 0,2435 | 0,0954 | 0,0481 | 0,0018 |
| S7P | 0,3620 | 0,0142 | 1,5680 | 0,2973 | 8,5832 | 1,3473 | 2,0371 | 0,0615 |
| SBP | 1,1581 | 0,1579 | 3,3526 | 0,5604 | 9,3673 | 0,6087 | 7,6416 | 1,3650 |
| Serine | 0,1103 | 0,0393 | 0,2730 | 0,0833 | 0,3400 | 0,0324 | 0,2609 | 0,1003 |
| Shikimate-3P | 0,1878 | 0,0560 | 0,1405 | 0,0366 | 0,1877 | 0,0067 | 0,1286 | 0,0155 |
| Succinate | 4,2137 | 0,8202 | 5,2332 | 0,2875 | 17,8272 | 1,1590 | 15,1605 | 1,4064 |
| Succinyl CoA | 0,1967 | 0,0572 | 0,0132 | 0,0041 | 0,0809 | 0,0097 | 0,1182 | 0,0215 |
| Threonine | 1,4490 | 0,2289 | 0,8979 | 0,2240 | 0,5818 | 0,0330 | 0,4858 | 0,1939 |
| Thymidine | 0,0016 | 0,0023 | 0,0228 | 0,0051 | 0,4321 | 0,1512 | 0,0241 | 0,0070 |
| TMP | 0,5485 | 0,1886 | 0,6294 | 0,1446 | 0,8461 | 0,1382 | 1,3342 | 0,3176 |
| Tryptophan | 0,0592 | 0,0014 | 0,0174 | 0,0070 | 0,0368 | 0,0123 | 0,0006 | 0,0012 |
| Tyrosine | 0,2172 | 0,0467 | 0,1395 | 0,0089 | 0,1464 | 0,0132 | 0,1411 | 0,0315 |
| UDP | 1,4382 | 0,0953 | 1,1286 | 0,1197 | 1,0863 | 0,1673 | 0,6604 | 0,0762 |
| UDP-Glc | 13,1398 | 1,4088 | 5,4908 | 1,0737 | 7,1119 | 0,8445 | 2,9441 | 0,9841 |
| UMP | 9,6123 | 2,1359 | 8,1389 | 0,6857 | 10,3661 | 0,8364 | 11,1597 | 1,7862 |
| Uridine | 1,1411 | 0,3153 | 3,5311 | 0,2001 | 10,3760 | 2,4885 | 0,6794 | 0,1428 |
| UTP | 1,8453 | 0,5262 | 1,2813 | 0,1211 | 1,1646 | 0,2005 | 3,9980 | 0,9592 |
| Valine | 0,2029 | 0,0169 | 0,0869 | 0,0062 | 0,2869 | 0,0764 | 0,1225 | 0,0289 |
| Xanthine | 0,0226 | 0,0085 | 0,0701 | 0,0063 | 0,0013 | 0,0013 | 0,0074 | 0,0034 |
| XMP | 0,1769 | 0,0398 | 0,4401 | 0,1175 | 0,8498 | 0,1281 | 1,6757 | 0,0129 |

Supplementary information: genomic sequence of *B. subtilis pgsBCAE*

ATGTGGTTACTCATTATAGCCTGTGCTGTCATACTGGTCATCGGAATATTAGAAAAACGA

CGACATCAGAAAAACATTGATGCCCTCCCTGTTCGGGTGAATATTAACGGCATCCGCGGA

AAATCGACTGTGACAAGGCTGACAACCGGAATATTAATAGAAGCCGGTTACAAGACTGTT

GGAAAAACAACAGGAACAGATGCAAGAATGATTTACTGGGACACACCGGAGGAAAAGCCG

ATTAAACGGAAACCTCAGGGGCCGAATATCGGAGAGCAAAAAGAAGTCATGAGAGAAACA

GTAGAAAGAGGGGCTAACGCGATTGTCAGTGAATGCATGGCTGTTAACCCAGATTATCAA

ATCATCTTTCAGGAAGAACTTCTGCAGGCCAATATCGGCGTCATTGTGAATGTTTTGGAA

GACCATATGGATGTCATGGGGCCGACGCTTGATGAAATTGCAGAAGCGTTTACTGCTACA

ATTCCTTATAATGGCCATCTTGTCATTACAGATAGTGAATATACCGAGTTCTTTAAACAA

AAAGCAAAAGAACGAAACACAAAAGTCATCATTGCTGATAACTCAAAAATTACAGATGAG

TATTTACGTAAATTTGAATACATGGTATTCCCTGATAACGCTTCTCTGGCGCTGGGTGTG

GCTCAAGCACTCGGCATTGACGAAGAAACAGCATTTAAGGGAATGCTGAATGCGCCGCCA

GATCCGGGAGCAATGAGAATTCTTCCGCTGATCAGTCCGAGCGAGCCTGGGCACTTTGTT

AATGGGTTTGCCGCAAACGACGCTTCTTCTACTTTGAATATATGGAAACGTGTAAAAGAA

ATCGGTTACCCGACCGATGATCCGATCATCATCATGAACTGCCGCGCAGACCGTGTCGAT

CGGACACAGCAATTCGCAAATGACGTATTGCCTTATATTGAAGCAAGTGAACTGATCTTA

ATCGGTGAAACAACAGAACCGATCGTAAAAGCCTACGAAGAAGGCAAAATTCCTGCAGAC

AAACTGCATGATCTAGAGTATAAGTCAACAGATGAAATTATGGAATTGTTAAAGAAAAGA

ATGCACAACCGTGTCATATATGGCGTCGGCAATATTCATGGTGCCGCAGAGCCTTTAATT

GAAAAAATCCACGAATACAAGGTAAAGCAGCTCGTAAGCTAGGGGGAAATGCAGACATGT

TCGGATCAGATTTATACATCGCACTAATTTTAGGTGTACTACTCAGTTTAATTTTTGCGG

AAAAAACAGGGATCGTGCCGGCAGGACTTGTTGTACCGGGATATTTAGGACTTGTGTTTA

ATCAGCCGGTCTTTATTTTACTTGTTTTGCTAGTGAGCTTGCTCACGTATGTCATTGTGA

AATACGGTTTATCCAAATTTATGATTTTGTACGGACGCAGAAAATTCGCTGCCATGCTGA

TAACAGGGATCGTCCTAAAAATCGCGTTTGATTTTCTATACCCGATTGTACCATTTGAAA

TCGCAGAATTTCGAGGAATCGGCATCATCGTGCCAGGTTTAATTGCCAATACCATTCAGA

AACAAGGTTTAACCATTACGTTCGGAAGCACGCTGCTATTGAGCGGAGCGACCTTTGCTA

TCATGTTTGTTTACTACTTAATTTAATGTAAGGTGTGTCAAACGATGAAAAAAGAACTGA

GCTTTCATGAAAAGCTGCTAAAGCTGACAAAACAGCAAAAAAAGAAAACCAATAAGCACG

TATTTATTGCCATTCCGATCGTTTTTGTCCTTATGTTCGCTTTCATGTGGGCGGGAAAAG

CGGAAACGCCGAAGGTCAAAACGTATTCTGACGACGTACTCTCAGCCTCATTTGTAGGCG

ATATTATGATGGGACGCTATGTTGAAAAAGTAACGGAGCAAAAAGGGGCAGACAGTATTT

TTCAATATGTTGAACCGATCTTTAGAGCCTCGGATTATGTAGCAGGAAACTTTGAAAACC

CGGTAACCTATCAAAAGAATTATAAACAAGCAGATAAAGAGATTCATCTGCAGACGAATA

AGGAATCAGTGAAAGTCTTGAAGGATATGAATTTCACGGTTCTCAACAGCGCCAACAACC

ACGCAATGGATTACGGCGTTCAGGGCATGAAAGATACGCTTGGAGAATTTGCGAAGCAAA

ATCTTGATATCGTTGGAGCGGGATACAGCTTAAGTGATGCGAAAAAGAAAATTTCGTACC

AGAAAGTCAACGGGGTAACGATTGCGACGCTTGGCTTTACCGATGTGTCCGGGAAAGGTT

TCGCGGCTAAAAAGAATACGCCGGGCGTGCTGCCCGCAGATCCTGAAATCTTCATCCCTA

TGATTTCAGAAGCGAAAAAACATGCGGACATTGTTGTTGTGCAGTCACACTGGGGACAAG

AGTATGACAATGATCCAAATGACCGCCAGCGCCAGCTTGCAAGAGCCATGTCTGATGCGG

GAGCTGACATCATCGTCGGCCATCACCCGCACGTCTTAGAACCGATTGAAGTATATAACG

GAACCGTCATTTTCTACAGCCTCGGCAACTTTGTCTTTGACCAAGGCTGGACGAGAACAA

GAGACAGTGCACTGGTTCAGTATCACCTGAAGAAAAATGGAACAGGACGCTTTGAAGTGA

CACCGATCGATATCCATGAAGCGACACCTGCGCCTGTGAAAAAAGACAGCCTTAAACAGA

AAACCATTATTCGCGAACTGACGAAAGACTCTAATTTCGCTTGGAAAGTAGAAGACGGAA

AACTGACGTTTGATATTGATCATAGTGACAAACTAAAATCTAAATAATCGGAGTGATAAA

GATGAAATTTGTCAAAGCCATCTGGCCGTTTGTTGCCGTAGCCATCGTGTTCATGTTTAT

GTCAGCTTTTAAATTCAATGATCAGCTGACAGATCAGGAAAAACAGAAGATTGATATGGA

AATGAATAAAATCCAGCAGCAGGAAGAACCGGTAAACGCCAATAAATAA
